# Manipulating terahertz phonon-polariton in the ultrastrong coupling regime with bound states in the continuum

**DOI:** 10.1038/s41377-025-02044-0

**Published:** 2025-10-09

**Authors:** Jiaxing Yang, Liyu Zhang, Kai Wang, Chen Zhang, Aoyu Fan, Zijian He, Zhidi Li, Xiaobo Han, Furi Ling, Peixiang Lu

**Affiliations:** 1 Wuhan National Laboratory for Optoelectronics and School of Physics, Huazhong University of Science and Technology, Wuhan, China; 2 School of Electronic and Information Engineering, Hubei University of Science and Technology, Xianning, China; 3 School of Optics and Electronic Information, Huazhong University of Science and Technology Wuhan, Wuhan, Hubei China; 4 Hubei Key Laboratory of Optical Information and Pattern Recognition, Wuhan Institute of Technology, Wuhan, China

**Keywords:** Terahertz optics, Polaritons

## Abstract

The strong coupling between photons and phonons in polar materials gives rise to phonon-polaritons that encapsulate a wealth of physical information, offering crucial tools for the ultrafast terahertz sources and the topological engineering of terahertz light. However, it is still quite challenging to form and manipulate the terahertz phonon-polaritons under the ultrastrong coupling regime till now. In this work, we demonstrate the ultrastrong coupling between the phonon (at 0.95 THz) in a MAPbI_3_ film and the metallic bound states in the continuum (BICs) in Au metasurfaces. The Rabi splitting can be continuously tuned from 28% to 48.4% of the phonon frequency by adjusting the parameters (size, shape and period) of Au metasurfaces, reaching the ultrastrong coupling regime. By introducing wavelet transform, the mode evolution information of the terahertz phonon-polariton is successfully extracted. It indicates that the phonon radiation intensity of the MAPbI_3_ film is enhanced as the coupling strength is increased. This work not only establishes a new platform for terahertz devices but also opens new avenues for exploring the intricate dynamics of terahertz phonon-polaritons.

## Abstract (web summary)

Metallic BIC metasurfaces enable ultrastrong coupling with perovskite phonons (48.4% Rabi) and enhanced THz phonon radiation, resolved via wavelet transform.

## Graphical abstract

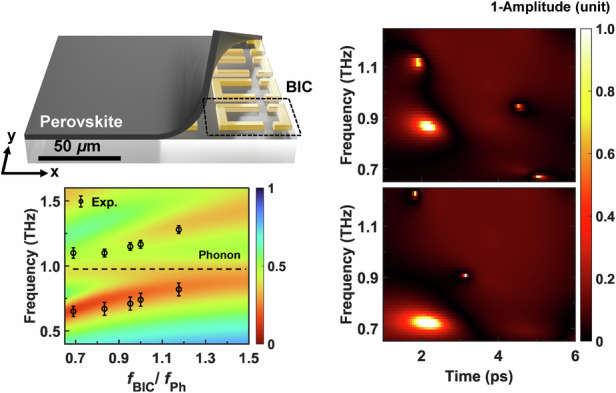

## Introduction

Phonon-polariton is the quasi-particle generated from the interaction between light and phonons in materials, which is widely distributed from mid-infrared to terahertz (THz) ranges^1–6^. It carries crucial physical information and exhibits potential applications, such as vacuum Bloch–Siegert shift^7^, topological control^8^, tunable laser sources^9^ and thermal emitters^2^. Notably, terahertz phonon-polariton is formed through the coupling of photons with THz phonons, which is a vital research focus within the realm of polaritons recently. Since the THz phonons widely exist in many prominent materials such as semiconductor quantum wells^10^, perovskites^11^, graphene^5^ and DNA molecules^12^, it can provide deep insights into these materials and crucial guidance for the design of terahertz sensors^13^ and detectors^14,15^. Therefore, it is essential to develop a hybrid system for THz phonon-polaritons with an enhanced coupling strength. The coupling strength in such systems is quantified by $\eta ={\Omega }_{R}/2{\omega }_{{\rm{Ph}}}$, where ${\Omega }_{R}$ represents the vacuum Rabi frequency and ${\omega }_{{\rm{Ph}}}$ the photon frequency. Especially for the hybrid system operating in the ultrastrong coupling (USC) regime (η > 0.1)^16–19^, it transcends conventional physical limits and exhibits unique phenomena^20–22^. The USC regime has garnered significant attention due to its demonstrated potential for modifying material properties, include modified electronic transport^23,24^, cavity chemistry^25,26^, or vacuum-field-induced super-conductivity^27,28^.

Most previous studies on terahertz phonon-polaritons are based on Fabry-Perot (F-P) cavities^29–33^ or plasmonic nanocavities^34–38^. Specifically, F-P cavities have a large mode volume with uniform local-field distributions, making it convenient for coupling with the thin films of organic molecules^32^ or crystalline semiconductor^39^. However, the local-field enhancement in F-P cavities is moderate, leading to the limitation in achieving ultrastrong coupling strength. Furthermore, it is still challenging for miniaturization and integration. In contrast, plasmonic nanocavities offer a stronger local-field in a much smaller mode volume in nanostructures^40^, thereby enhancing the coupling strength of the system. However, the typical mode volume of plasmonic nanocavities is much smaller than crystalline particles, such as MAPbI_3_ (~300 nm)^41^, α-lactose (>10 *μ*m)^42^, leading to a spatial mismatching between the cavity and the phonon modes. As mentioned, both cavities show the limitations for further enhancing the coupling strength of THz phonon-polaritons. Therefore, it is urgent to develop a new hybrid system for studying THz phonon-polaritons under the ultrastrong coupling regime.

In recent years, bound states in the continuum (BICs) have been actively studied in optical systems^43,44^, accompanied by many discoveries and applications such as optical microcavities^45^, lasers^46^ and sensors^47,48^. BICs have been shown the excellent abilities for enhancing light-matter interactions^49,50^, providing a strongly enhanced local-field, high quality factor ($Q$), ultrathin thickness, and broad resonance tunability via variation of their geometrical parameters^51^. In particular, BICs can reduce thermal losses while simultaneously increasing coupling strength in the system of metallic metasurfaces^52,53^. These capabilities make metallic metasurfaces based on BICs a promising platform for achieving and modulating ultrastrong coupling in the terahertz range. In addition, MAPbI_3_ is a perovskite material with excellent optoelectronic properties, which shows potential applications in batteries^54^, solar energy conversion^55^, and light-emitting diodes (LEDs)^56^. MAPbI_3_ exhibits strong phonon vibrations at 0.95 THz, and can be easily crystallized into a high-quality crystalline film through spin-coating and thermal annealing, making it a highly significant terahertz material that has attracted considerable attention^57–60^. Therefore, MAPbI_3_ is a suitable phonon material for THz phonon-polaritons.

Current investigations of USC in terahertz phonon-polariton systems predominantly rely on Fourier-transform spectroscopy to characterize stationary spectral anti-crossing features. However, this approach obscures critical dynamical information, particularly the real-time mode evolution and phase-resolved intensity variations in phonon-polaritons. Such limitations obstruct the experimental exploration of dynamics process in USC systems. Wavelet analysis emerges as a powerful mathematical framework for resolving localized time-frequency characteristics of non-stationary signals. This technique captures joint temporal-spectral information using scalable, translatable wavelet basis functions, making it suitable for probing transient processes in USC systems. The generalized Morse wavelet transform is a parametrically optimized analytic wavelet, which has demonstrated exceptional performance in telecommunications and biomedical signal processing. This methodology can provide multidimensional insights into terahertz polariton systems, including coupling-strength modulation phase-discontinuity and other critical parameters which cannot be observed through conventional spectral integration techniques.

Here, we report the demonstration of the ultrastrong coupling between the phonon in MAPbI_3_ film and the metallic BICs in Au metasurfaces. The unit cell of the Au metasurface consists of the coupled C-shaped Au split ring resonator (SRR) pairs, forming the BIC modes. The resonance linewidth of BICs is controlled via the asymmetry of the unit cell, matching with the damping rate of phonon vibration. By varying the size of unit cells, the BIC resonance frequency is continuously tuned to match with the MAPbI_3_ phonon frequency at 0.95 THz, and a Rabi splitting up to 0.28 THz is obtained. Importantly, the Rabi splitting can further be tuned from 28% to 48.4% of the phonon frequency by precisely controlling the mode volume of BICs, reaching the ultrastrong coupling regime. By introducing wavelet transform, the mode evolution information of the terahertz phonon-polariton is successfully extracted. It indicates that the phonon radiation intensity of the MAPbI_3_ film is enhanced as the coupling strength is increased. Our results open up new possibilities for the control of polaritons and reveal new information within the phonon-polaritons system.

## Results

### Sample design and BICs theory

**Fig. 1:**
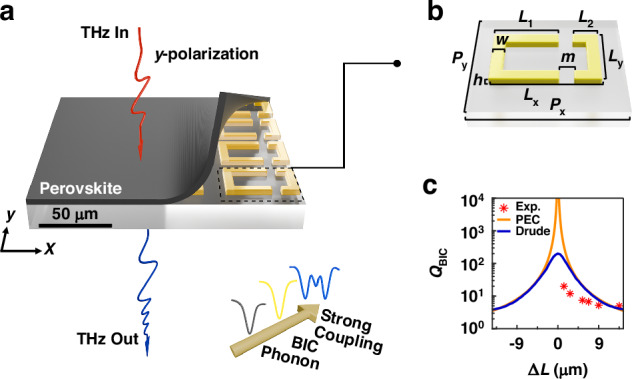
The structure of Au-MAPbI_3_ metasurface and the properties of BIC. **a** Illustration of Au metasurfaces covered with a MAPbI_3_ thin film. **b** The structure of the BIC unit. The geometrical unit cell parameters are: ${P}_{{\rm{x}}}$ = 66 *μ*m, ${P}_{{\rm{y}}}$ = 32 *μ*m, ${L}_{{\rm{x}}}$ = 50 *μ*m, ${L}_{{\rm{y}}}$ = 25 *μ*m, ${L}_{1}$ = 39.5 *μ*m, $m$ = 5 *μ*m, $w$ = 5 *μ*m, $h$ = 200 nm, with the period $d={P}_{{\rm{x}}}-{L}_{{\rm{x}}}$. The tuning of the resonance position of the BIC modes is realized by introducing multiplicative scaling factor *S*, which scales the geometrical parameters of metasurfaces by multiplying all parameters by *S*. Terahertz waves pass through the sample in y-polarization and emerge from the quartz substrate side. (bottom right) Schematic diagram of strong coupling between phonons and BICs. **c** Quality factor ($Q$) of the BIC modes varies with the degree of asymmetry. Red stars represent experimental data, while blue crosses represent the Drude model results, and simulations using parameters employed in the experiment. The orange solid line represents the ideal PEC case. The degree of asymmetry is defined by the expression $\Delta L=$*L*_1_-*L*_*2*_

Figure 1a illustrates the proposed hybrid system, which is composed of Au metasurface and MAPbI_3_ film (see Method for details). Figure 1b shows the unit cell of the Au metasurface, it can be seen the asymmetric C-shaped Au SRR pairs, and the asymmetric degree can be controlled by the difference of arm lengths, $\Delta L={L}_{1}-{L}_{2}$. As the two electromagnetic modes in Au SRR pairs are coupled with each other, two new modes are generated. In special cases, one of these modes exhibits zero radiation loss, which is called BIC. The metallic BIC modes discussed in this paper can be elucidated using the Friedrich–Wintgen (F-W) BICs theory. In principle, BIC modes are analyzed by treating the metal as a perfect electric conductor (PEC), the simulated $Q$ factor exhibits an inverse square relationship with $\Delta L$ as shown by the orange curve in Fig. 1c. For symmetric structures ($\Delta L=0$) the resonance vanishes as the quasi-BIC turns into a true BIC, i.e., the $Q$ factor of the BIC modes approaches infinity, because the loss associated with the metal is considered to be zero in the PEC model. When the Drude model is used to depict the refractive index of Au, the simulated $Q$ factor of the BIC modes is shown by the blue curve in Fig. 1c, which matches well with the PEC model when $\varDelta L$ is large. However, as $\varDelta L$ approaches zero, the $Q$ factor is limited to below 200, because of the inherent losses of Au in the Drude model. The red stars in Fig. 1c represent the experimental results of the $Q$ factor which show a trend consistent with the Drude model (see Method for experiment setup). The relationship approximately follows an inverse square proportionality with respect to $\varDelta L$. Due to factors such as uncertainties during the sample preparation, the measured $Q$ values are lower than the simulated results under the Drude model at low asymmetry (see Note 1, Supplementary Information).

MAPbI_3_ is selected as the phonon material for the coupling system. In the classical model, the dielectric constant dispersion of phonon modes is typically described by Lorentz model. The relative permittivity can be expressed as^11^:

1 $${\varepsilon }_{{\rm{r}}}={\varepsilon }_{\infty }+\chi ={\varepsilon }_{\infty }-\mathop{\sum }\limits_{m}\frac{{{{S}_{0m}* \omega }_{0{\rm{m}}}}^{2}}{{\omega }^{2}-{{\omega }_{0{\rm{m}}}}^{2}+i\omega {\gamma }_{m}}$$

where $\chi$ is the polarization susceptibility, ${\varepsilon }_{\infty }$ is the permittivity at infinite frequency, ${\omega }_{0}$ and $\gamma$ represent the phonon frequency and damping rate. The refractive index is given by $n=\sqrt{{\varepsilon }_{{\rm{r}}}}$ (see Note 2, Supplementary Information).

### Strong coupling in Au-MAPbI_3_ hybrid metasurfaces

**Fig. 2:**
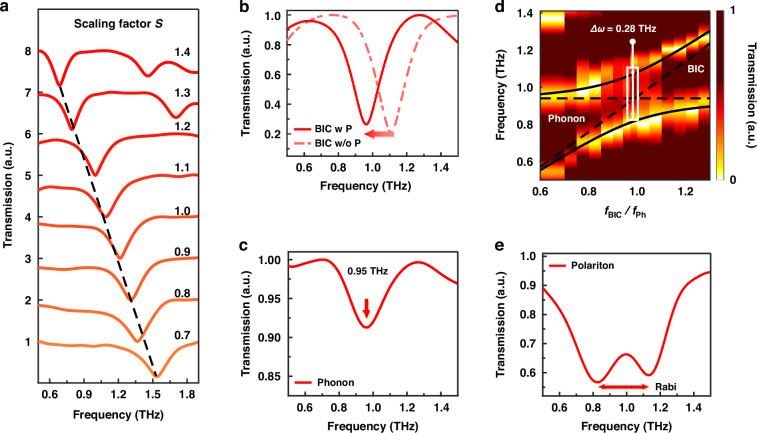
The transmission spectra of the BIC metasurfaces before and after spin-coating of perovskite, as well as the pure perovskite film. **a** Measured transmission amplitudes of BIC resonances across the frequency of perovskite phonon tuned via scaling the in-plane geometric parameters. The new parameters were derived by multiplying *S* with the in-plane geometric parameters. ${P}_{{\rm{xnew}}}=S{P}_{{\rm{x}}}$, ${P}_{{\rm{ynew}}}=S{P}_{{\rm{y}}}$, ${L}_{{\rm{xnew}}}=S{L}_{{\rm{x}}}$, ${L}_{{\rm{ynew}}}=S{L}_{{\rm{y}}}$, ${L}_{1{\rm{new}}}=S{L}_{1}$, ${m}_{{\rm{new}}}={Sm}$, ${w}_{{\rm{new}}}={Sw}$. **b** Transmission amplitude of the Au metasurface coated with an amorphous perovskite film is represented by red solid line, while that of pare Au metasurface without perovskite is represented by a red dished line. **c** The transmission amplitude of a MAPbI_3_ film spin-coated on a quartz substrate. The arrow indicates the frequency of phonon resonance. **d** Transmission amplitude of Au-MAPbI_3_ metasurfaces for different scaling factors shows a characteristic and anticrossing mode pattern close to the MAPbI_3_ phonon. **e** Transmission amplitude of Au metasurface coated with a crystallized perovskite film while the resonance position of BICs is tuned to 0.95 THz, which reveals a Rabi splitting of 0.28 THz

As shown in Fig. 2a, the BIC resonance peak is continuously tuned from 0.68 to 1.53 THz through the phonon frequency at 0.95 THz by adjusting the scaling factor *S* of the unit cell. $S$ parameter is defined as the proportional magnified ratio in size in comparison with the standard size of the unit cell in Fig. 1b. Noting that the asymmetric parameter is kept as the constant of $\varDelta L$ = 34 *μ*m, when the resonance linewidth of the BIC mode is matched with the damping rate of the phonon. At this parameter of asymmetry, the Rabi splitting due to the coupling between the BICs and phonons is most pronounced (see Note 3, Supplementary Information). Figure 2b shows the position of the BIC resonance peak before (red dashed line) and after (red solid line) MAPbI_3_ film coating. It shows a significant redshift after coating, due to the influence of the vacuum dielectric constant of MAPbI_3_. At this stage, the MAPbI_3_ film has not undergone annealing and is in an intermediate phase, which exhibits no phonon resonance. Figure 2c shows the transmission amplitude of the crystallized MAPbI_3_ film on a quartz substrate after thermal annealing. The quality of crystallization of perovskite significantly affects the coupling strength. The MAPbI_3_ thin film has a thickness of approximately 200 nanometers and exhibits good crystallization quality, with a particle size significantly smaller than the minimum size of the metasurface unit cells. This allows for optimal coupling between the BICs and the phonons of MAPbI_3_ (Figure S19a, Supplementary Information).

After crystallization of the perovskite films on the Au metasurfaces, as shown in Fig. 2d, the transmission amplitude of the system exhibits a pronounced anti-crossing feature. To explain the changes in modes before and after coupling between BICs and phonons, the variations in the energy levels of the system and the interactions between these energy levels were analyzed. From the Hamiltonian of the system, the energy levels and interactions that govern the dynamics of the coupled modes within the hybrid metasurfaces can be derived. The full Hamiltonian can be expressed in the following form^10^:

2 $$\hat{H}={\hat{H}}_{\mathrm{BIC}}+{\hat{H}}_{\mathrm{Ph}}+{\hat{H}}_{\mathrm{int}}+{\hat{H}}_{\mathrm{dia}}$$

${\hat{H}}_{{\rm{BIC}}}$ and ${\hat{H}}_{{\rm{Ph}}}$ are the bare photon and phonon Hamiltonians, respectively. ${\hat{H}}_{\mathrm{int}}$ is the light-matter interaction term, where the coupling strength is represented by $g$. ${\hat{H}}_{{\rm{dia}}}$ is the ${A}^{2}$ term, which is the quadratic term of the vector potential A of the light field. Diagonalizing this Hamiltonian via a 4×4 matrix $M$, where $M\vec{V}=\omega \vec{V}$ and $\det \left|M-\omega I\right|=0$, yields the polariton eigenfrequencies ${\omega }_{\pm }$ (see Note 3, Supplementary Information). The resonance frequency of BIC and phonon are *ω*_BIC_ and ${\omega }_{{\rm{Ph}}}$, and damping rate of BIC and phonon are ${\gamma }_{{\rm{BIC}}}$ = 0.18 THz and ${\gamma }_{{\rm{P}}h}$ = 0.20 THz, the properties of the BICs and phonons are extracted from measurement (Fig. 2b and c). And $g$ is the coupling strength between the two coupled elements. The corresponding Rabi splitting is defined as ${\Omega }_{{\rm{R}}}$ by substituting ${\omega }_{+}$ − ${\omega }_{-}$. As shown in Fig. 2d, the interaction between the BICs and the phonons leads to a hybridization effect, resulting in the emergence of new resonant peaks that are characteristic of phonon-polaritons. The transmission amplitude shows clear anticrossing behaviour, which is an essential feature of strong coupling systems. It indicates that the BICs and phonons have strongly coupled to form new phonon-polariton modes. The transmission amplitude of the polariton modes shown in Fig. 2d was analyzed according to Eq. 2. The minimum of the splitting corresponds precisely to the condition where ${\omega }_{{\rm{BIC}}}$ = ${\omega }_{{\rm{Ph}}}$ = 0.95 THz (Fig. 2e). This result indicates that the system’s Rabi splitting is 0.28 THz, which is correspond to 1.15 meV. According to established criteria of strong coupling^61^ $c=2g/\sqrt{({\gamma }_{{\rm{BIC}}}^{2}+{\gamma }_{{\rm{Ph}}}^{2})/2} > 1$, which leads to $c$ = 1.47 >1. Obviously, the experimental results substantially exceed the required conditions, confirming that the coupling strength of our Au-MAPbI_3_ hybrid metasurfaces indeed reach the criteria of strong coupling. Furthermore, the experimental results align well with the full Hamiltonian results (Figure S6, Supplementary Information), providing additional validation for our findings. The formation of these phonon-polariton modes enhances the light-matter interaction, leading to increased field localization and modified dispersion characteristics.

### Tailoring the ultrastrong coupling in Au-MAPbI_3_ hybrid metasurfaces

The general definition of Rabi splitting^16^ is

3 $${\hslash \Omega }_{{\rm{R}}}=\hslash {\rm{\Omega }}\sqrt{N}\propto \sqrt{\frac{N}{{V}_{\mathrm{eff}}}}$$

where $N$ is the number of oscillators participating in the coupling, and $\hslash \Omega$ represents the contribution from each oscillator. In strong coupling systems, the coupling strength is increased as the mode volume of BIC (see Note 4, Supplementary Information) decreases according to Eq. 3. In situations where it is challenging to increase the maximum electric field strength, compressing the mode volume by reducing the period of the metasurface can effectively enhance the Rabi splitting. So, the metasurfaces are designed with different periods by varying the parameter $d={P}_{{\rm{x}}}-{L}_{{\rm{x}}}$ to tune the coupling strength by changing the mode volume. A series of different parameters *d* from 50 to 2 *μ*m are selected based on the metasurface with $d$ = 16 *μ*m in Fig. 2d. ${P}_{{\rm{y}}}-{L}_{{\rm{y}}}$ is scaled proportionally with *d*, while other geometrical parameters of the metasurface remain constant according to the structural parameters from Fig. 1b. In this case, the resonance of the BIC modes can match with phonons, which are in the optimal position for generating phonon-polaritons.

**Fig. 3:**
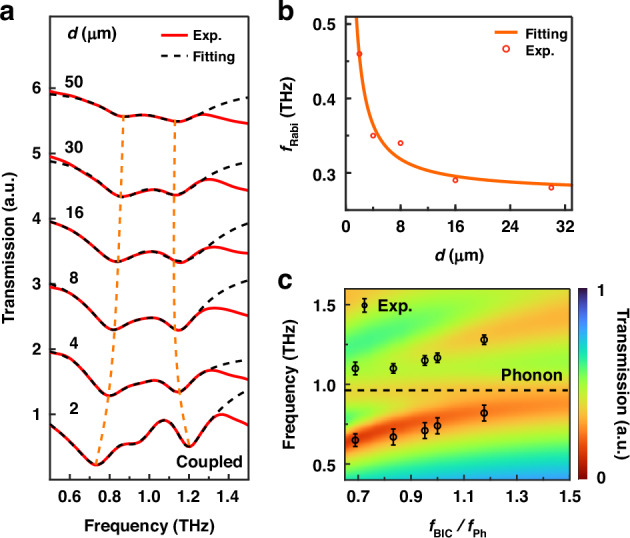
Tunable BIC-phonon coupling strength via geometric scaling. **a** Transmission amplitude that corresponds to the on-resonance case with the period $d$ changed, where ${L}_{{\rm{x}}}$ = 50 *μ*m is constant. The red curve represents the experimental data, while the dark dashed line corresponds to the fit obtained from the experimental data. The peaks of polariton are connected by orange dashed lines. **b** Experimentally measured correlation between Rabi splitting and parameter *d*, showing monotonic enhancement of splitting magnitude with decreasing *d*. **c** The transmission spectra evolution with BIC cavity resonance frequency. The color map represents simulation results implementing both BIC configuration and dual-phonon perovskite oscillator parameters (0.95 + 1.85 THz), while dark markers denote experimentally extracted peak positions of the upper and lower polariton branches

The transmission amplitude of the samples is shown in Fig. 3a. When the period of the metasurface increases as *d* increases from 16 to 50 *μ*m, the Rabi splitting is modulated from 0.28 THz to 0.26 THz, indicating a slight change. When *d* is less than 16 *μ*m, the Rabi splitting is significantly enhanced as *d* decreases. Specifically, as *d* is reduced from 16 to 2 *μ*m, the Rabi splitting is modulated from 0.28 THz to 0.46 THz. As a result, the Rabi splitting of the phonon-polariton is increased as *d* decreases, with a modulation range from 28% to 48.4% of the phonon frequency. By extracting the peak frequencies of two branches from each transmission spectrum, the relationship between Rabi splitting and $d$ can be fitted. By simulation the electric near-fields in one unit cell with Lumerical, the mode volume (${V}_{{\rm{eff}}}$) was calculated as a function of the parameter $d$ (2 *μ*m -16 *μ*m). (see Note 4, Supplementary Information). Figure S7 quantitatively establishes the inverse proportionality between mode volume ${V}_{{\rm{eff}}}$ and interlayer spacing *d*, showing a 62% reduction in ${V}_{{\rm{eff}}}$ as *d* decreases from 16 *μ*m to 2 *μ*m. This trend aligns with the *d*-dependent variation in Rabi splitting intensity shown in Fig. 3b. In addition, given that $d$ is larger than the particle size observed after the crystallization of the MAPbI_3_, the number of participating phonons remains at saturation levels, which means the influence of the number of phonons can be neglected. Besides, we demonstrate that BIC asymmetry exerts minimal influence on Rabi splitting values in phonon-polariton systems due to intrinsic-loss-dominated electric field enhancement limitations and high-order BIC mode in metallic metasurfaces, despite the strong coupling criterion parameter *c* increasing from 1.40 to 2.24 (see Note 5, Supplementary Information). Therefore, it is sufficient only to focus on the relationship between the Rabi splitting and mode volume ${V}_{{\rm{eff}}}$.

To obtain the tunability of the phonon-polariton in the metasurface with the maximum Rabi splitting, 5 samples are prepared based on the metasurfaces with $d$ = 2 *μ*m. The resonance of BIC modes is tuned from 0.7 to 1.2 THz by changing the scaling factor $S$. Figure 3c presents the pseudo-color map derived from simulations incorporating both the BIC configuration and dual-phonon perovskite oscillator parameters (0.95 + 1.85 THz), with dark markers indicating experimentally extracted peak positions of the upper and lower energy branches. Clearly, anticrossing curves (color map in Fig. 3c) were obtained by simulation, which means the frequency of phonon-polariton is tunable. The minimum frequency difference between the two polariton branches is 0.46 THz, occurring at the position where ${\omega }_{{\rm{BIC}}}={\omega }_{{\rm{Ph}}}$. Consequently, the Rabi splitting produced by the sample is 48.4% of ${\omega }_{{\rm{Ph}}}$, placing the system well within the regime of ultrastrong coupling. This value represents one of the highest coupling strengths achieved in terahertz phonon-polariton systems to date.

While excellent agreement exists between experimental measurements and numerical simulations, the observed position of the upper energy branch deviates from theoretical predictions based on the single-phonon (0.95 THz) Hamiltonian. This inconsistency suggests the presence of additional coupling mechanisms beyond the initial model. Detailed analysis in the Note 6 (Supplementary Information) indicates significant perturbation from the 1.85 THz phonon mode, which exhibits the spectral overlap with the coupled system^62^. Theory and simulations incorporating both phonons yield Rabi splitting values closer to experimental observations, confirming the necessity of concerning 1.85 THz phonon.

### Wavelet analysis process

The wavelet transform is a powerful time-frequency analysis tool that overcomes the inherent trade-off between temporal and spectral resolution in conventional Fourier transforms. Unlike Fourier analysis, which decomposes a signal into infinite sinusoidal waves, wavelet transform uses localized basis functions (“wavelets”) scaled and shifted across the time domain. This enables precise resolution of transient features in non-stationary signals like THz pulses. We employed the generalized Morse wavelet^63^. The generalized Morse wavelet in the Fourier domain is^64^

4 $${\psi }_{\beta ,\gamma }\left(\omega \right)=U\left(\omega \right){a}_{\beta ,\gamma }{\omega }^{\beta }{e}^{-{\omega }^{\gamma }}$$

where $U(\omega )$ is the unit step function, ${a}_{\beta ,\gamma }$ is a normalizing constant, $\beta$ controls the time-domain decay rate (related to compactness), and $\gamma$ characterizes the symmetry of the Morse wavelet.

Wavelet analysis yields the instantaneous spectrum for each delay in the mode evolution time trace, providing balanced results in both time and frequency domains. This method can be introduced as a powerful tool for analyzing the temporal information of THz emission and detection.

### Time evolution of electric mode via wavelet analysis

**Fig. 4:**
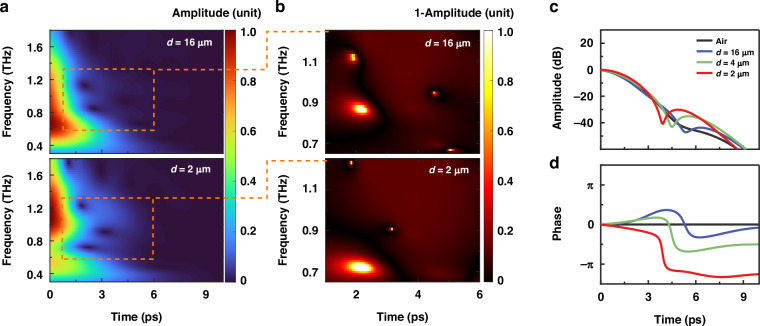
Time-resolved terahertz field dynamics via wavelet analysis. **a** Time-evolved spectrum was obtained through the wavelet transform method with $d$ = 16 *μ*m, $d$ = 2 *μ*m. **b** Time-frequency domain maps of the regions $d$ = 16 *μ*m and 2 *μ*m after background removal within the orange dashed boxes. Time evolution of (**c**) intensity and (**d**) phase of the terahertz field at ${\omega }_{{\rm{Ph}}}$ = 0.95 THz for different $d$ parameters

To analyze the modes evolution of the generated phonon-polariton in this strong coupling system, THz fields passing through metasurfaces with representative periods are selected for the wavelet transform. As depicted in Fig. 4a, the instantaneous spectrums of metasurfaces with four different parameters $d$ are generated by wavelet analysis (details see Fig. S13, Supplementary Information), which is performed with the generalized Morse wavelet^63^ with *β* = 60, *γ* = 3 (see Note 7, Supplementary Information). The zero delay corresponds to the peak electric field of the terahertz wave prior to coupling with the material. The depth information in Fig. 4a represents the absolute amplitude of the normalized THz field, showing the frequency distribution at different times as the THz wave passes through the metasurface. The amplitude of the THz field attenuates to zero in 10 ps, indicating the rapid decay time for the polariton modes. This observation aligns with the classical expression for mode evolution $Q=2\pi t/2T={\omega }_{0}t/2$, where $t$ represents the oscillator lifetime, consistent with the damping rate derived from the results in Fig. 3a. The modes in the directly obtained time-frequency spectrum are not clear enough, due to the influence of THz waves that do not participate in coupling. Therefore, in Fig. 4b, clear modes evolution spectrum of the phonon-polariton are obtained by subtracting the backgrounds of Fig. 4a. The three highlighted areas in Fig. 4b, from top to bottom represent the upper polariton branch, the phonon mode, and the lower polariton branch respectively. The highlighted mode frequencies in Fig. 4b correspond directly to the positions of the valley in the transmission amplitude spectrum in Fig. 3a.

Notably, Fig. 4a contain time evolution information of modes that cannot be obtained from the frequency domain spectrum through the Fast Fourier Transform (FFT). From the comparison of Fig. 4b, it can be observed that the phonon mode appears 2 ps earlier as *d* decreases. To further analyze the reasons for the temporal variations of the phonon modes in different samples, as shown in Fig. 4c,d, the intensity and phase evolution information at *ω* = 0.95 THz are obtained by processing the time-frequency spectrums for different parameters $d$. It is observed notable changes in the phase of THz field in Fig. 4d. Specifically, both the THz field intensity and phase of phonon mode exhibit abrupt transitions, with the timing of these transitions occurring earlier as $d$ decreases. Furthermore, the wavepacket after the transition is primarily attributed to phonon re-emission (see Note 8, Supplementary Information). By comparing the THz field intensity of the re-emitted phonon portion in Fig. 4c, it can be observed that the phonon radiation from the hybrid metasurface with parameter $d$ = 2 *μ*m is 10 dB higher than that with $d$ = 50 *μ*m. It is concluded that as $d$ decreases, the coupling strength increases, and the radiative intensity of the phonon increases by 10 dB. This highlights the essence of enhanced coupling strength, which involves maximizing the proportion of radiative losses in the presence of material dissipation while minimizing the proportion of absorption losses. Similarly, the phase of the upper and lower polariton branches is significantly changed evolved over time, which reveals the process of energy absorption and re-emission within strong coupling systems (Fig. S15, Supplementary Information).

**Fig. 5:**
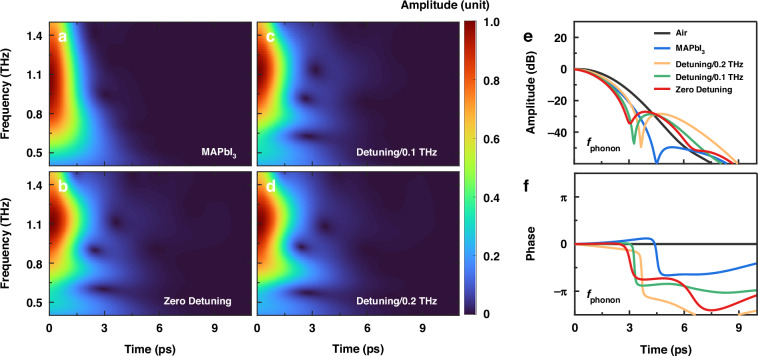
Detuning-dependent polariton-phonon coupling and field evolution at the phonon frequency. **a**–**d** Wavelet transform analysis of time-domain signals reconstructed from incident terahertz wavepackets and three coupled decaying oscillators, corresponding to the upper polariton branch, lower polariton branch, and phonon mode. Time-frequency maps are normalized to the incident pulse amplitude. **a** Bare MAPbI_3_ thin film. **b** Zero detuning. **c** Finite detuning ($\Delta =0.1$ THz). **d** Finite detuning ($\Delta =0.2$ THz). Time evolution of (**e**) intensity and (**f**) phase of the terahertz field at ${\omega }_{{\rm{Ph}}}$ = 0.95 THz for air, bare MAPbI_3_ and hybrid metasurface at the finite detuning with the same *d* parameter

To verify the role of photon-phonon hybridization, we conducted experiments at finite detuning ($\Delta =\mathrm{0,0.1,0.2}$ THz) while maintaining $d=2$ *µ*m. As shown in Fig. 5, detuning significantly reduced the delayed phonon re-emission intensity (by ~5 dB). In contrast, the hybrid system at zero detuning exhibited 20 dB stronger emission than bare perovskite films, confirming enhanced radiative efficiency via polariton. At zero detuning, complete hybridization channels energy exchange between the BIC mode and phonon, maximizing radiative losses. Conversely, detuning disrupts resonance, localizing energy in lossy phonon modes and increasing absorption losses. This behavior underscores the coherent coupling mechanism inherent in the phonon-polariton system, where the interactions between the phonons and the polaritons lead to the formation of these two modes. This behavior underscores the coherent coupling mechanism inherent in the phonon-polariton system, where the interactions between the phonons and the polaritons lead to the formation of these two modes.

## Discussion

In summary, we demonstrate the ultrastrong coupling in Au-MAPbI_3_ hybrid metasurfaces based on metallic BICs. The Rabi splitting can be continuously adjusted from 28% to 48.4% of the phonon frequency by modifying the period of the Au metasurfaces, thus reaching the ultrastrong coupling regime. Through wavelet transform, we successfully extracted the mode evolution information of the terahertz phonon-polariton. It indicates that the phonon radiation intensity of the MAPbI_3_ film is enhanced with increasing coupling strength. Our findings provide critical insights into the interaction between the metallic BICs and the phonons of perovskite. The methodologies and insights gained can be applied to explore multi-mode coupling phenomena across various platforms and materials. In addition, Perovskite materials exhibit intrinsic semiconductor properties, making them promising candidates for optoelectronic applications. This perovskite-based platform demonstrates significant potential for further optoelectronic modulation in ultrastrong coupling regime^20,65^, with promising applications in topological engineering^30^, ultrafast modulator^66^ and electronic transport^67^. This research fosters the development of innovative photonic devices, and opens new pathways for future investigations into polaritons and strong coupling effects in the terahertz regime.

## Materials and methods

### Sample fabrication

The Au metasurfaces, with a thickness of 200 nm, were fabricated on a quartz substrate using UV lithography and Electron Beam Evaporation (EBE). The prepared samples were subsequently subjected to treatment in an ultraviolet ozone cleaner to thoroughly eliminate surface organic contaminants. Perovskite thin films were created using the solution spin coating method (Fig. S18, Supplementary Information). A mixture of MAI and PbI_2_ was dissolved in a solution of N, N-dimethylformamide (DMF) and dimethyl sulfoxide (DMSO) in a 1:1 ratio. The solution was stirred on a magnetic stirrer at 60°C for 12 hours until fully dissolved. The solution was then filtered to obtain the MAPbI_3_ perovskite precursor solution. Then, 40 *μ*L of the MAPbI_3_ precursor solution was spin-coat onto the metasurface at 1000 rpm for the first 10 seconds, followed by 6000 rpm for the next 30 seconds. 100 *μ*L of chlorobenzene was dispensed onto the surface quickly and evenly. After spin coating, the sample was placed on a hot plate and annealed at 100°C for 15 minutes to complete the perovskite thin film preparation about 250 nm (see Fig. S19, Supplementary Information). Lead iodide (PbI_2_) was purchased from the Tokyo Chemical Industry. Methylammonium iodide (MAI) was purchased from MaterWin company. N, N-Dimethylformamide (DMF), dimethyl sulfoxide (DMSO), and chlorobenzene (CB) were obtained from Aladdin.

### Experiment setup

We conducted terahertz spectroscopy measurements using our independently developed terahertz time-domain spectroscopy (THz-TDS) system (Fig. S20, Supplementary Information), and obtained detailed information about the terahertz field through Fast Fourier Transform (FFT) and wavelet analysis. The THz-TDS system is driven by a Ti-sapphire femtosecond laser, which generates laser pulses with a duration of 35 fs, a repetition rate of 1 kHz, and a center wavelength of 800 nm. The femtosecond laser beam is split into two paths, used for terahertz generation and detection respectively. We utilize efficient metallic spintronic emitters of ultra-broadband terahertz radiation to generate terahertz pulses based on the principle of ultrafast photoinduced spin currents^68^. The emitted terahertz pulse is polarized in the y-direction in Fig. 1b, and passes through the sample before simultaneously impinges on a ZnTe crystal with another femtosecond laser pulse, where the detection of the terahertz pulse is accomplished via the electro-optic effect. To eliminate the impact of water molecules in the air on the experiment, the portion of the test system through which terahertz waves propagate is filled with dry air maintained at a humidity level below 5%.

## Supplementary information

Supplementary Information for Manipulating terahertz phonon-polariton in the ultrastrong coupling regime with bound states in the continuum

## Data Availability

The main data supporting the findings of this study are available within the article and its Supplementary Information files. Extra data are available from the corresponding author upon reasonable request.

## References

[1] Barra-Burillo, M. et al. Microcavity phonon polaritons from the weak to the ultrastrong phonon–photon coupling regime. *Nat. Commun.* **12**, 6206 (2021). 34707119 10.1038/s41467-021-26060-x PMC8551273

[2] Lu, G. Y. et al. Engineering the spectral and spatial dispersion of thermal emission via polariton–phonon strong coupling. *Nano Lett.* **21**, 1831–1838 (2021). 33587855 10.1021/acs.nanolett.0c04767

[3] Mekonen, S. M. et al. Coupled metamaterial–phonon terahertz range polaritons in a topological insulator. *ACS Photonics* **11**, 2242–2246 (2024).

[4] Martínez-Martínez, L. A. et al. Can ultrastrong coupling change ground-state chemical reactions?. *ACS Photonics* **5**, 167–176 (2018).

[5] Epstein, I. et al. Far-field excitation of single graphene plasmon cavities with ultracompressed mode volumes. *Science* **368**, 1219–1223 (2020). 32527826 10.1126/science.abb1570

[6] Wan, W. W., Yang, X. D. & Gao, J. Strong coupling between mid-infrared localized plasmons and phonons. *Opt. Express* **24**, 12367–12374 (2016). 27410151 10.1364/OE.24.012367

[7] Li, X. W. et al. Vacuum Bloch–Siegert shift in Landau polaritons with ultra-high cooperativity. *Nat. Photonics* **12**, 324–329 (2018).

[8] Kim, S. et al. Topological control of 2D perovskite emission in the strong coupling regime. *Nano Lett.* **21**, 10076–10085 (2021). 34843262 10.1021/acs.nanolett.1c03853

[9] Ohtani, K. et al. An electrically pumped phonon-polariton laser. *Sci. Adv.* **5**, eaau1632 (2019). 31309138 10.1126/sciadv.aau1632 PMC6625821

[10] Zhang, Q. et al. Collective non-perturbative coupling of 2D electrons with high-quality-factor terahertz cavity photons. *Nat. Phys.* **12**, 1005–1011 (2016).

[11] La-o-vorakiat, C. et al. Phonon mode transformation across the orthohombic–tetragonal phase transition in a lead iodide perovskite CH_3_NH_3_PbI_3_: a terahertz time-domain spectroscopy approach. *J. Phys. Chem. Lett.* **7**, 1–6 (2016). 26633131 10.1021/acs.jpclett.5b02223

[12] Cheon, H. et al. Terahertz molecular resonance of cancer DNA. *Sci. Rep.* **6**, 37103 (2016). 27845398 10.1038/srep37103 PMC5109182

[13] Odit, M. et al. Observation of supercavity modes in subwavelength dielectric resonators. *Adv. Mater.* **33**, 2003804 (2021). 10.1002/adma.202003804 33169472

[14] Wang, R. D. et al. Ultrasensitive terahertz biodetection enabled by quasi-BIC-based metasensors. *Small* **19**, 2301165 (2023). 10.1002/smll.202301165 37162455

[15] Ferguson, B. & Zhang, X. C. Materials for terahertz science and technology. *Nat. Mater.* **1**, 26–33 (2002). 12618844 10.1038/nmat708

[16] Frisk Kockum, A. et al. Ultrastrong coupling between light and matter. *Nat. Rev. Phys.* **1**, 19–40 (2019).

[17] Askenazi, B. et al. Ultra-strong light–matter coupling for designer Reststrahlen band. *N. J. Phys.* **16**, 043029 (2014).

[18] Askenazi, B. et al. Midinfrared ultrastrong light–matter coupling for THz thermal emission. *ACS Photonics* **4**, 2550–2555 (2017).

[19] Canales, A. et al. Polaritonic linewidth asymmetry in the strong and ultrastrong coupling regime. *Nanophotonics* **12**, 4073–4086 (2023). 39635646 10.1515/nanoph-2023-0492 PMC11501566

[20] Günter, G. et al. Sub-cycle switch-on of ultrastrong light–matter interaction. *Nature* **458**, 178–181 (2009). 19279631 10.1038/nature07838

[21] Scalari, G. et al. Ultrastrong coupling of the cyclotron transition of a 2D electron gas to a THz metamaterial. *Science* **335**, 1323–1326 (2012). 22422976 10.1126/science.1216022

[22] Huber, R. et al. How many-particle interactions develop after ultrafast excitation of an electron–hole plasma. *Nature* **414**, 286–289 (2001). 11713523 10.1038/35104522

[23] Paravicini-Bagliani, G. L. et al. Magneto-transport controlled by Landau polariton states. *Nat. Phys.* **15**, 186–190 (2019).

[24] Orgiu, E. et al. Conductivity in organic semiconductors hybridized with the vacuum field. *Nat. Mater.* **14**, 1123–1129 (2015). 26366850 10.1038/nmat4392

[25] Chikkaraddy, R. et al. Single-molecule strong coupling at room temperature in plasmonic nanocavities. *Nature* **535**, 127–130 (2016). 27296227 10.1038/nature17974 PMC4947385

[26] Thomas, A. et al. Tilting a ground-state reactivity landscape by vibrational strong coupling. *Science* **363**, 615–619 (2019). 30733414 10.1126/science.aau7742

[27] Schlawin, F., Cavalleri, A. & Jaksch, D. Cavity-mediated electron-photon superconductivity. *Phys. Rev. Lett.* **122**, 133602 (2019). 31012600 10.1103/PhysRevLett.122.133602

[28] Thomas, A. et al. Exploring superconductivity under strong coupling with the vacuum electromagnetic field. *J. Chem. Phys.* **162**, 134701 (2025). 40166996 10.1063/5.0231202

[29] Di Virgilio, L. et al. Controlling the electro-optic response of a semiconducting perovskite coupled to a phonon-resonant cavity. *Light Sci. Appl.* **12**, 183 (2023). 37491336 10.1038/s41377-023-01232-0 PMC10368682

[30] Ergoktas, M. S. et al. Topological engineering of terahertz light using electrically tunable exceptional point singularities. *Science* **376**, 184–188 (2022). 35389774 10.1126/science.abn6528 PMC7612901

[31] Damari, R. et al. Strong coupling of collective intermolecular vibrations in organic materials at terahertz frequencies. *Nat. Commun.* **10**, 3248 (2019). 31324768 10.1038/s41467-019-11130-y PMC6642260

[32] Kim, H. S. et al. Mechanical control of polaritonic states in lead halide perovskite phonons strongly coupled in THz microcavity. *J. Phys. Chem. Lett.* **14**, 10318–10327 (2023). 37943739 10.1021/acs.jpclett.3c02717

[33] Gao, W. L. et al. Continuous transition between weak and ultrastrong coupling through exceptional points in carbon nanotube microcavity exciton–polaritons. *Nat. Photonics* **12**, 362–367 (2018).

[34] Shelton, D. J. et al. Strong coupling between nanoscale metamaterials and phonons. *Nano Lett.* **11**, 2104–2108 (2011). 21462937 10.1021/nl200689z

[35] Jin, X. et al. Reshaping the phonon energy landscape of nanocrystals inside a terahertz plasmonic nanocavity. *Nat. Commun.* **9**, 763 (2018). 29472554 10.1038/s41467-018-03120-3 PMC5823850

[36] Jaber, A. et al. Hybrid architectures for terahertz molecular polaritonics. *Nat. Commun.* **15**, 4427 (2024). 38789427 10.1038/s41467-024-48764-6 PMC11126624

[37] Roh, Y. et al. Ultrastrong coupling enhancement with squeezed mode volume in terahertz nanoslots. *Nano Lett.* **23**, 7086–7091 (2023). 37471630 10.1021/acs.nanolett.3c01913

[38] Kim, H. S. et al. Phonon-polaritons in lead halide perovskite film hybridized with THz metamaterials. *Nano Lett.* **20**, 6690–6696 (2020). 32786930 10.1021/acs.nanolett.0c02572

[39] Bajoni, D. et al. Polariton laser using single micropillar GaAs − GaAlAs semiconductor cavities. *Phys. Rev. Lett.* **100**, 047401 (2008). 18352332 10.1103/PhysRevLett.100.047401

[40] Zhang, Z. Y. et al. Ultrastrong coupling between THz phonons and photons caused by an enhanced vacuum electric field. *Phys. Rev. Res.* **3**, L032021 (2021).

[41] Ding, J. X. et al. Design growth of MAPbI_3_ single crystal with (220) facets exposed and its superior optoelectronic properties. *J. Phys. Chem. Lett.* **9**, 216–221 (2018). 29271206 10.1021/acs.jpclett.7b03020

[42] Patel, S. R. & Murthy, Z. V. P. Effect of process parameters on crystal size and morphology of lactose in ultrasound-assisted crystallization. *Cryst. Res. Technol.* **46**, 243–248 (2011).

[43] Hsu, C. W. et al. Bound states in the continuum. *Nat. Rev. Mater.* **1**, 16048 (2016).

[44] Cong, L. Q. & Singh, R. Symmetry-protected dual bound states in the continuum in metamaterials. *Adv. Optical Mater.* **7**, 1900383 (2019).

[45] Wang, W. H. et al. Brillouin zone folding driven bound states in the continuum. *Nat. Commun.* **14**, 2811 (2023). 37198151 10.1038/s41467-023-38367-y PMC10192215

[46] Che, Y. et al. Ultrasensitive photothermal switching with resonant silicon metasurfaces at visible bands. *Nano Lett.* **24**, 576–583 (2024). 37970822 10.1021/acs.nanolett.3c03288 PMC10798257

[47] Tan, T. C. et al. Active control of nanodielectric-induced THz quasi-BIC in flexible metasurfaces: a platform for modulation and sensing. *Adv. Mater.* **33**, 2100836 (2021). 10.1002/adma.202100836 34050568

[48] Ren, Z. H. et al. Overcoming high-quality limitations in plasmonic metasurfaces for ultrasensitive terahertz applications. *ACS Nano* **18**, 21211–21220 (2024). 39079002 10.1021/acsnano.4c04565

[49] Weber, T. et al. Intrinsic strong light-matter coupling with self-hybridized bound states in the continuum in van der Waals metasurfaces. *Nat. Mater.* **22**, 970–976 (2023). 37349392 10.1038/s41563-023-01580-7 PMC10390334

[50] Lim, W. X. et al. Ultrafast all-optical switching of germanium-based flexible metaphotonic devices. *Adv. Mater.* **30**, 1705331 (2018). 10.1002/adma.201705331 29327454

[51] Kang, M. et al. Applications of bound states in the continuum in photonics. *Nat. Rev. Phys.* **5**, 659–678 (2023).

[52] Liang, Y., Tsai, D. P. & Kivshar, Y. From local to nonlocal high-*Q* plasmonic metasurfaces. *Phys. Rev. Lett.* **133**, 053801 (2024). 39159090 10.1103/PhysRevLett.133.053801

[53] Yang, F. et al. Bending sensing based on quasi bound states in the continuum in flexible terahertz metasurface. *Adv. Optical Mater.* **11**, 2300909 (2023).

[54] Koh, T. M. et al. Formamidinium tin-based perovskite with low *E*_g_ for photovoltaic applications. *J. Mater. Chem. A* **3**, 14996–15000 (2015).

[55] Liu, J. L. et al. Electron injection and defect passivation for high-efficiency mesoporous perovskite solar cells. *Science* **383**, 1198–1204 (2024). 38484055 10.1126/science.adk9089

[56] Kojima, A. et al. Organometal halide perovskites as visible-light sensitizers for photovoltaic cells. *J. Am. Chem. Soc.* **131**, 6050–6051 (2009). 19366264 10.1021/ja809598r

[57] Zhizhchenko, A. et al. Single-mode lasing from imprinted halide-perovskite microdisks. *ACS Nano* **13**, 4140–4147 (2019). 30844247 10.1021/acsnano.8b08948

[58] Zhizhchenko, A. Y. et al. Light-emitting nanophotonic designs enabled by ultrafast laser processing of halide perovskites. *Small* **16**, 2000410 (2020). 10.1002/smll.202000410 32309903

[59] Onoda-Yamamuro, N., Matsuo, T. & Suga, H. Dielectric study of CH_3_NH_3_PbX_3_ (X = Cl, Br, I). *J. Phys. Chem. Solids* **53**, 935–939 (1992).

[60] Li, Y. F. et al. Ultrabroadband, ultraviolet to terahertz, and high sensitivity CH_3_NH_3_PbI_3_ perovskite photodetectors. *Nano Lett.* **20**, 5646–5654 (2020). 32609527 10.1021/acs.nanolett.0c00082

[61] Zhang, L. et al. Photonic-crystal exciton-polaritons in monolayer semiconductors. *Nat. Commun.* **9**, 713 (2018). 29459736 10.1038/s41467-018-03188-x PMC5818602

[62] Kim, D. et al. Cavity-mediated superthermal phonon correlations in the ultrastrong coupling regime. Preprint 10.48550/arXiv.2409.04505 (2024).

[63] Olhede, S. C. & Walden, A. T. Generalized Morse wavelets. *IEEE Trans. Signal Process.* **50**, 2661–2670 (2002).

[64] Lilly, J. M. jLab: a data analysis package for Matlab, v.1.7.3. (2024). http://www.jmlilly.net/code URL.

[65] Anappara, A. A. et al. Controlling polariton coupling in intersubband microcavities. *Superlattices Microstructures* **41**, 308–312 (2007).

[66] Malerba, M. et al. Ultrafast (≈10 GHz) mid-IR modulator based on ultrafast electrical switching of the light–matter coupling. *Appl. Phys. Lett.* **125**, 041101 (2024).

[67] Pisani, F. et al. Electronic transport driven by collective light-matter coupled states in a quantum device. *Nat. Commun.* **14**, 3914 (2023). 37400430 10.1038/s41467-023-39594-z PMC10318076

[68] Seifert, T. et al. Efficient metallic spintronic emitters of ultrabroadband terahertz radiation. *Nat. Photonics* **10**, 483–488 (2016).

