# Supplementary material for: Manipulating terahertz phonon-polariton in the ultrastrong coupling regime with bound states in the continuum
Source: Light Sci Appl. 2025 Oct 9;14:360. doi: 10.1038/s41377-025-02044-0 (PMC12511402; doi:10.1038/s41377-025-02044-0)
Supplement: Supplementary file 1 — Supplementary Information for Manipulating terahertz phonon-polariton in the ultrastrong coupling regime with bound states in the continuum [file 41377_2025_2044_MOESM1_ESM.docx]

**Supplementary Information for**

**Manipulating terahertz phonon-polariton in the ultrastrong coupling regime with bound states in the continuum**

*Jiaxing Yang^1#^, Liyu Zhang^1#^, Kai Wang^1,2*^, Chen Zhang^1^, Aoyu Fan^3^, Zijian He^1^, Zhidi Li^1^, Xiaobo Han^4^, Furi Ling^3*^, Peixiang Lu^1,4*^*

^1^Wuhan National Laboratory for Optoelectronics and School of Physics, Huazhong University of Science and Technology, Wuhan 430074, China

^2^School of Electronic and Information Engineering, Hubei University of Science and Technology, Xianning 437100, China

^3^Hubei Key Laboratory of Optical Information and Pattern Recognition, Wuhan Institute of Technology, Wuhan 430205, China

^4^School of Optics and Electronic Information, Huazhong University of Science and Technology Wuhan, Hubei 430074, P.R. China

*Corresponding authors:

#These authors contributed equally to this work.

[kale_wong@hust.edu.cn](mailto:kale_wong@hust.edu.cn) (KW), [lingfuri@mail.hust.edu.cn](mailto:lingfuri@mail.hust.edu.cn) (FRL), [lupeixiang@hust.edu.cn](mailto:lupeixiang@hust.edu.cn) (PXL)

**Contents:**

**Supplementary Note 1: Formation of Metallic BIC**

**Supplementary Note 2: Drude-Lorentz Model for Perovskite Phonon**

**Supplementary Note 3: Hopfield Model Theory for Strong Coupling**

**Supplementary Note 4: Relationship between Mode Volume and Rabi Splitting**

**Supplementary Note 5: Rabi Splitting at Different BIC Asymmetry**

**Supplementary Note 6: Impact of 1.85 THz Phonon Mode of MAPbI_3_**

**Supplementary Note 7: Wavelet Transform**

**Supplementary Note 8: Evolution of Phonon-polariton**

**Supplementary Note 9: Sample Fabrication**

**Supplementary Note 10: Experiment Setup**

**Supplementary Note 1:** **Formation of Metallic BIC**

Figure S1a shows the eigenmode of the arrays of single C-shaped split ring resonator (SRR). The field intensity is stronger at the outer edges, especially at the endpoints of the SRR, which facilitates the coupling of modes depicted in Figure S2. As shown in Figure S1b, when the arm length $L_{1}$ decreases, the eigenfrequency increases linearly, while the loss is approximately constant.


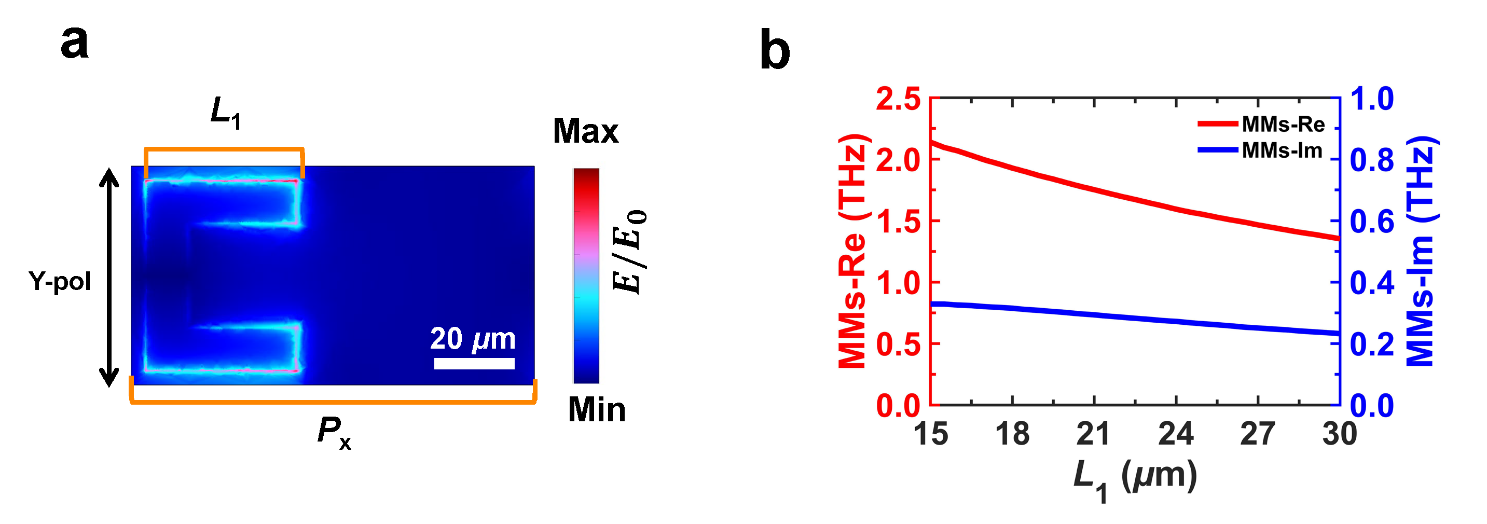


**Figure S1 | a,** The electric field distribution of the eigenmode of SRR. **b,** Eigenfrequency of the eigenmode of different arm lengths.


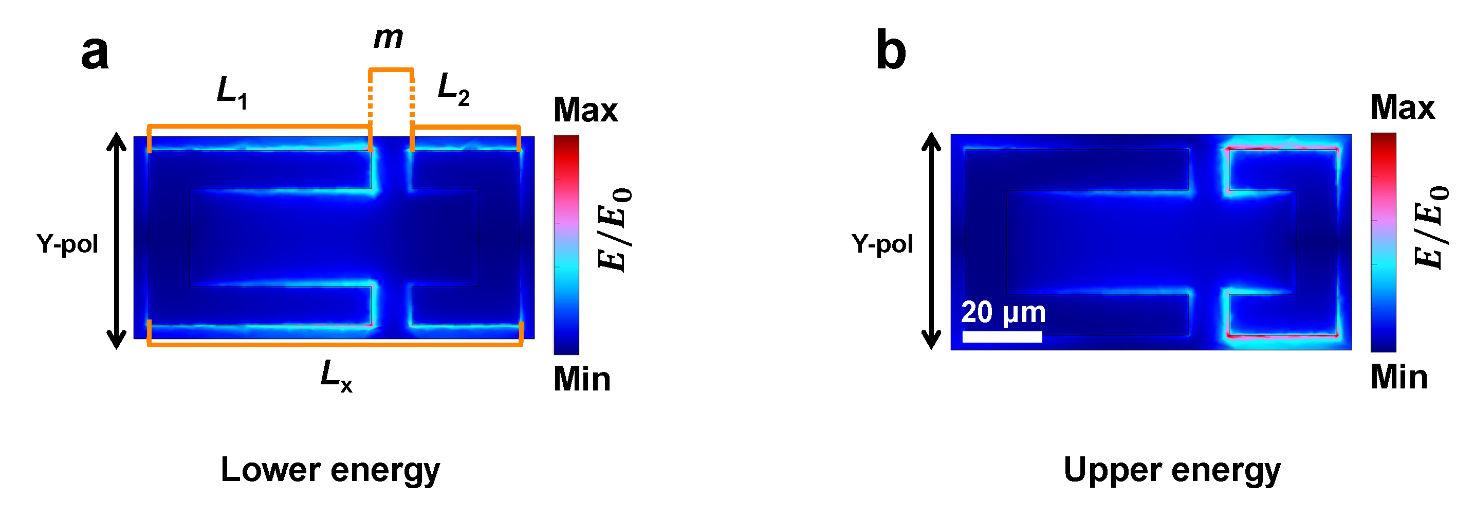


**Figure S2 | a-b,** The electric field distribution of the two generated eigenmodes in the asymmetrical metasurfaces. **(a)** lower branch, **(b)** upper branch.

Next, two C-shaped SRRs are placed opposite to each other, the modes are coupled by nearfield. The gap length *m* between them is fixed to ensure the coupling between the two modes remains relatively constant. Figures S2a, b depict the field distributions of the low-frequency mode (lower band) and the high-frequency mode (upper band) on the left and the right respectively. It is noted that the field intensity of the low-frequency mode primarily concentrates on one side of the structure with the longer arms, while the high-frequency mode exhibits the opposite behavior.

The coupling of two modes in the unit of the metasurface can be elucidated through a Hamiltonian^1,2^,

$\begin{aligned} H=\left( \begin{matrix} \omega_{1}-i\gamma_{1i} & \kappa\\ \kappa& \omega_{2}-i\gamma_{2i} \end{matrix} \right)-i\left( \begin{matrix} \gamma_{1r} & \sqrt{\gamma_{1r}\gamma_{2r}} \\ \sqrt{\gamma_{1r}\gamma_{2r}} & \gamma_{2r} \end{matrix} \right)\# MACROBUTTON MTPlaceRef \backslash* MERGEFORMAT SEQ MTEqn \backslash h \backslash* MERGEFORMAT (S SEQ MTEqn \backslash c \backslash* Arabic \backslash* MERGEFORMAT 1) \end{aligned}$ where $\kappa$ is the interaction potential between the two modes, $\omega_{1}, \omega_{2}$ are the resonant frequencies, $\gamma_{1i},\gamma_{2i}$ and $\gamma_{1r} ,\gamma_{2r}$ are nonradiative and radiative damping rates of the two modes respectively, $\sqrt{\gamma_{1r}\gamma_{2r}}$ represents the interference of radiation.

When metal is treated as perfect electric conductor (PEC), the non-radiative damping rate is zero. The eigenfrequencies of the two new hybrid modes are

$\begin{aligned} \omega_{u,d}=\left[ \omega_{1}+\omega_{2}+i\left( \gamma_{1r}+\gamma_{1r} \right) \right]/2 \\ \pm\sqrt{\left( \frac{\omega_{1}+\omega_{2}+i\left( \gamma_{1r}+\gamma_{2r} \right)}{2} \right)^{2}+\left[ \kappa^{2}-\omega_{1}\omega_{2}+i\left( \gamma_{2r}\omega_{1}+2\kappa\sqrt{\gamma_{1r}\gamma_{2r}}+\gamma_{1r}\omega_{2} \right) \right]}\#(S2) \end{aligned}$

Therefore, when

$$\begin{aligned} \kappa\left( \gamma_{1r}-\gamma_{2r} \right)=\sqrt{\gamma_{1r}\gamma_{2r}}\left( \omega_{1}-\omega_{2} \right)\#\left( S3 \right) \end{aligned}$$

one eigenvalue is real and the eigenmode turns into a BIC, while the damping rate of the other eigenmode doubles. Apparently, when the unit has side-to-side symmetry, Equation (S3) holds.

Figures S3a, b illustrate the eigenmode analysis, where the metal is considered as PEC. The theory and simulations are in good agreement. The eigenfrequencies exhibit an anticrossing behavior, and the damping rate of the lower modes approaches zero at the high symmetry point. This coupling behavior has been experimentally validated in Figure S3c.

Figures S4a and b correspond to the evolution of the mode's $Q$-factor in parameter space when considering the metal as PEC and as Drude-Lorentz material^3^. The asymmetry parameter is defined by^4^

$$\begin{aligned} \delta=\frac{L_{1}-L_{2}}{L_{1}}\#\left( S4 \right) \end{aligned}$$

When radiation losses are significant, absorption losses can be neglected. However, when the lower branch mode approaches BIC, absorption losses must be taken into account. For PEC, this mode's $Q$-factor can exceed $10^{5}$, whereas with the Drude model, the $Q$-factor peaks around 200. Near this high-symmetry point, radiation loss follows a parabolic trend, while the $Q$-factor adheres to the well-known inverse square relationship.

$$Q=\omega/{2\gamma}$$

$\begin{aligned} Q\left( \delta\right)\propto\delta^{-2}\#\left( S5 \right) \end{aligned}$

This phenomenon can be observed in the experiment in Figure S4c.

The experimental Q-factors in Fig 1c were extracted via Fano fitting of the transmission spectra in Figure S4c using:

$$\begin{aligned} T\left( \omega\right)=T_{0}\frac{\left( q+\epsilon\right)^{2}}{1+\epsilon^{2}},\epsilon=\frac{\omega-\omega_{BIC}}{\gamma_{BIC}/2} \#(S6) \end{aligned}$$

where$\gamma_{BIC}$ (damping rate) directly determines$Q=\omega_{BIC}/\gamma_{BIC}$ ​.

Figure S4d shown Drude-model-based simulations of transmission spectra for varying Δ*L*. While experimental Fano line shapes are less pronounced at small Δ*L* due to finite time-domain resolution (~25 ps), the simulated spectra exhibit clear Fano line shapes (dashed curves), corroborating the experimental trend of decreasing Q-factor with increasing Δ*L*, as shown in Figure S4c. These complementary observations confirm the self-consistency between simulated and measured data.


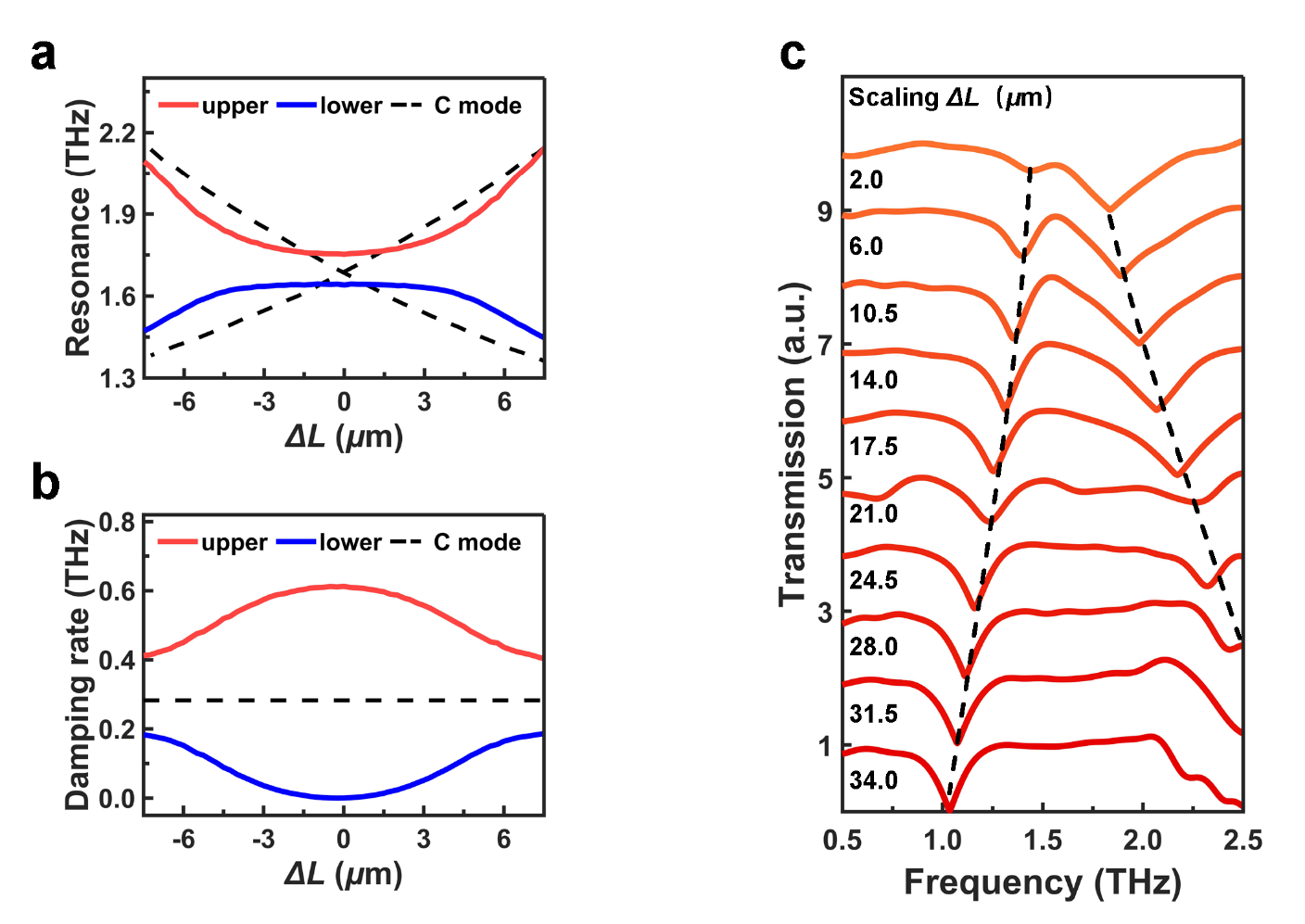


**Figure S3 | a-b,** The effect of coupling on the resonant frequencies and losses of two eigenmodes in simulation. **(a)** Resonant frequencies **(b)** Losses. **c,** The effect of coupling on the resonant frequencies and losses of two modes in the experiment**.**


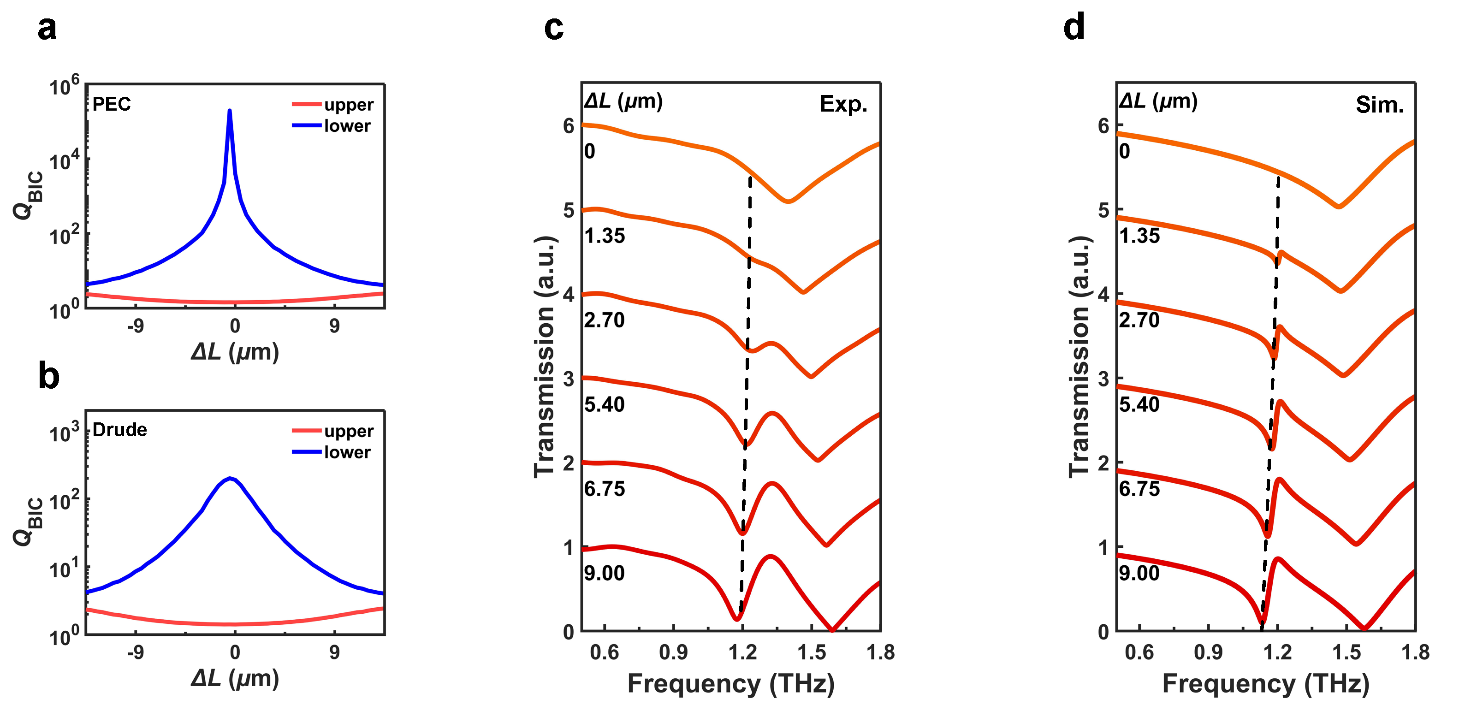


**Figure S4 | a-b,** Simulated $Q$ factor of the BIC mode varies with the degree of symmetry breaking. **(a)** metal as PEC, **(b)** metal as Drude-Lorentz material. **c,** Transmission amplitudes for different symmetry-broken BIC metasurfaces. **d,** Drude-model-based simulations of transmission spectra for varying Δ*L.*

**Supplementary Note 2:** **Drude-Lorentz Model for Perovskite Phonon**

We utilize the Drude-Lorentz model with two poles to describe the phonons in perovskites^5^,

$$\begin{aligned} \varepsilon_{r}=\varepsilon_{\infty}+\chi=\varepsilon_{\infty}-\sum_{m} \frac{{{S_{0m}*\omega}_{0m}}^{2}}{\omega^{2}-{\omega_{0m}}^{2}+i\omega\gamma_{m}}\#\left( S7 \right) \end{aligned}$$

where $\varepsilon_{\infty}$ = 4.2 is the background permittivity, $\omega_{01}$= 0.95 THz and $\omega_{02}$= 1.85 THz are the resonance frequencies,$\gamma_{1}$ = 0.20 THz and $\gamma_{2}$ = 0.59 THz are the damping rates, $S_{01}$= 1.81 and $S_{02}$= 2.18 are the oscillator strength^6^.

While we attempted to extract permittivity directly from THz transmission data (Figure 2c), uncertainties arise from finite film thickness uniformity (±15 nm) and experimental conditions, as shown in Figure S5b. We acknowledge the challenges in precise permittivity extraction and emphasize that the provided parameters are consistent with literature benchmarks.


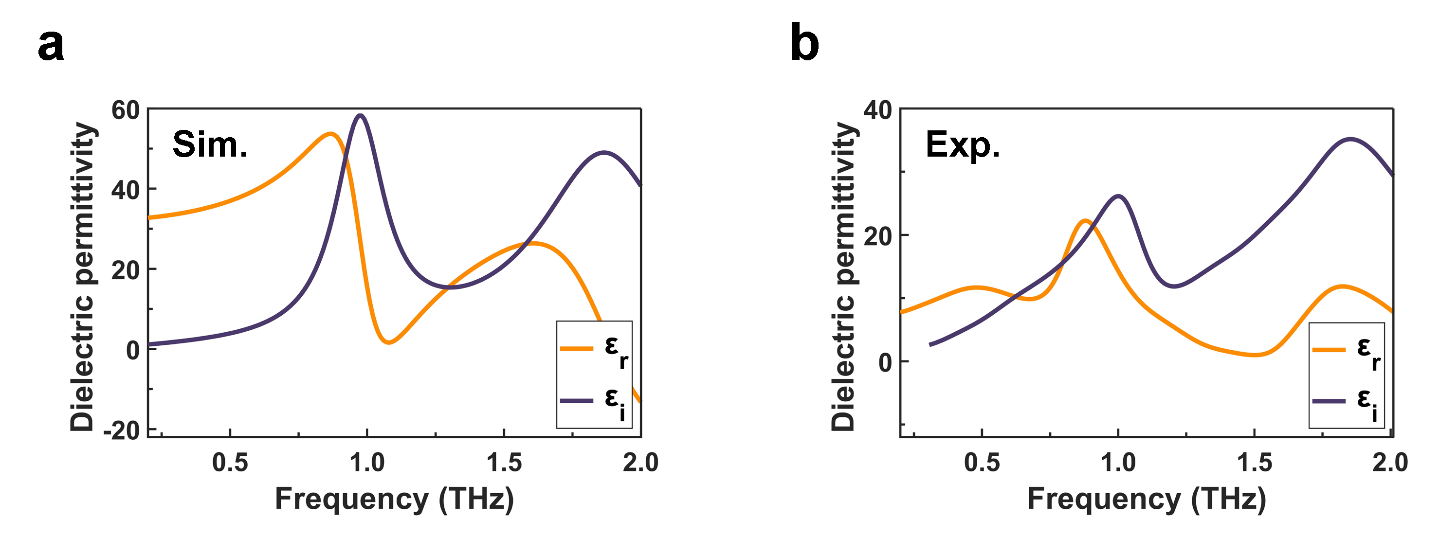


**Figure S5 | a,** Dielectric permittivity of MAPbI_3_ using the Lorentz model. **b,** Dielectric permittivity of MAPbI_3_ extracted from THz transmission data.

**Supplementary Note 3:** **Hopfield Model Theory for Strong Coupling**

We revised the analysis using the Hopfield-Bogoliubov framework to rigorously model USC effects. The coupled system full Hamiltonian can be expressed as^7^:

$$\begin{aligned} \hat{H}=\hat{H}_{\mathrm{BIC}}+\hat{H}_{\mathrm{Ph}}+\hat{H}_{\mathrm{int}}+\hat{H}_{\mathrm{dia}}\#\left（ S8 \right） \end{aligned}$$

$$\begin{aligned} \hat{H}_{\mathrm{BIC}}= \hbar\omega_{\text{BIC}}a^{\dagger}a \end{aligned}$$

$$\begin{aligned} \hat{H}_{\mathrm{Ph}}= \hbar\omega_{\text{Ph}}b^{\dagger}b \end{aligned}$$

$$\begin{aligned} \hat{H}_{\mathrm{int}}= \hbar g\left( a^{\dagger}+a \right)\left( b^{\dagger}-b \right) \end{aligned}$$

$$\begin{aligned} \hat{H}_{\mathrm{dia}}=\frac{\hbar g^{2}}{\omega_{\mathrm{Ph}}}\left( a^{\dagger}+a \right)\left( a^{\dagger}+a \right) \end{aligned}$$

where$a^{\dagger}$,$a$and $b^{\dagger}$, $b$are the creation/annihilation operators for the BIC and phonon modes, respectively. Diagonalizing this Hamiltonian via a 4×4 matrix $\mathbf{M}$, where$\mathbf{M}\vec{\mathbf{V}}=\omega\vec{\mathbf{V}}$ and $\det\left| \mathbf{M-}\omega\mathbf{I} \right|=0$, yields the polariton eigenfrequencies $\omega_{\pm}$.


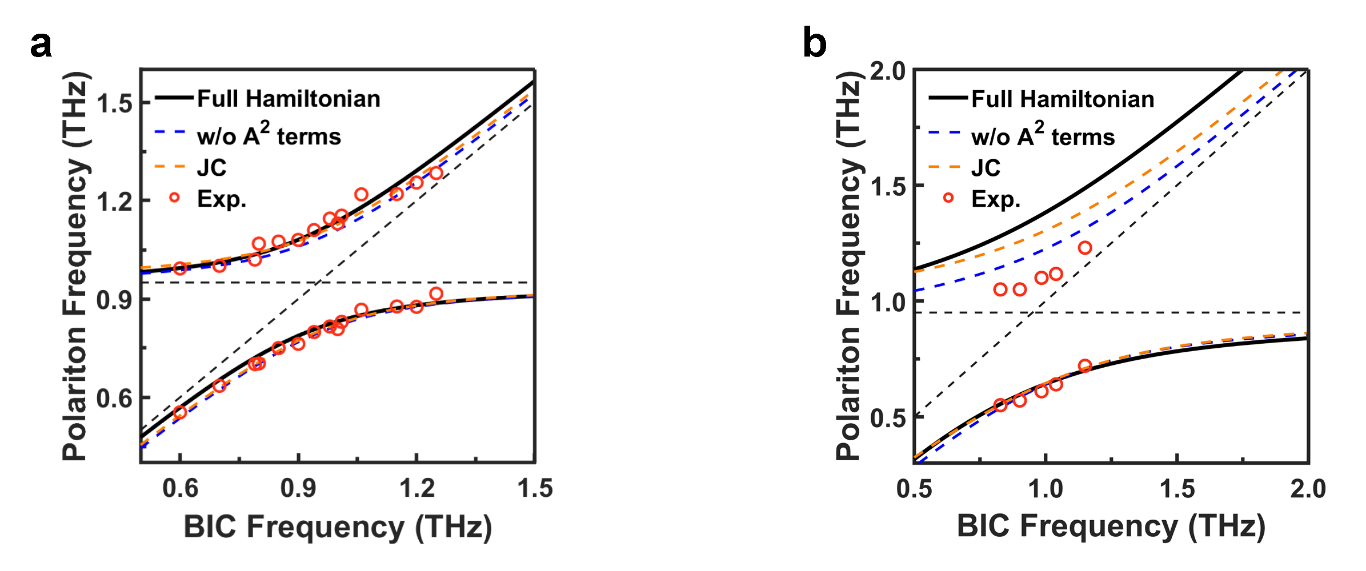


**Figure S6 |** The fits to our experimental data for the first mode using different Hamiltonians: the full Hamiltonian (black curves), the JC model (orange curves) and without $A^{2}$ terms (blue curves). The full Hamiltonian reproduced the experimental data well, suggesting the non-negligible contribution of the counter-rotating terms on the polariton frequencies, which confirms the system is entry into the ultrastrong coupling regime. **(a)** The fit was achieved when $g/\omega_{\mathrm{ph}}$ = 0.15 for $d$ =16 *μ*m (measured Rabi splitting $\Omega_{R}=$ 0.28 THz). **(b)** The fit was achieved when $g/\omega_{\mathrm{ph}}$ = 0.30 for $d$ =2 *μ*m (measured Rabi splitting $\Omega_{R}=$ 0.46 THz).

As shown in Figures S6a, b (black curves), the full Hopfield model predicts asymmetric for the upper and lower polaritons a hallmark of USC ^8^ while the RWA-based JC model (orange curves) fails to capture this asymmetry. The revised theory aligns quantitatively with experimental data (red dots). The fit in Figure S6a was achieved when $g/\omega_{\mathrm{ph}}$ = 0.15 for $d$ = 16 *μ*m (measured Rabi splitting $\Omega_{R}=$ 0.28 THz) and the fit in Figure S6b was achieved when $g/\omega_{\mathrm{ph}}$ = 0.30 for $d$ =2 *μ*m (measured Rabi splitting $\Omega_{R}=$ 0.46 THz).

To assess the contributions of both the counter-rotating terms (CRT) and $A^{2}$ terms, we systematically analyzed the polariton spectra using different theoretical models. we have performed comprehensive fitting of our experimental data using three different models (Figure S6). In Figure S6a (Rabi splitting = 0.28 THz), all models yield similar results. However, in the ultrastrong coupling regime (Figure S6b, Rabi splitting = 0.46 THz), we observe discrepancies in fitting the upper polariton branch, which we attribute to the influence of higher-order modes (high-frequency phonons and metasurface modes). As discussed in Supplementary Note 4, we have developed a more complete theoretical framework incorporating four-mode coupling, which provides excellent agreement between theory and experiment.

**Supplementary Note 4: Relationship between Mode Volume and Rabi Splitting**

We conducted systematic numerical simulations to quantitatively analyze the dependence of mode volume on structural parameters in our BIC metasurface. We simulated the electric near-fields in one unit cell with Lumerical, the mode volume ($V_{\mathrm{eff}}$) was calculated as a function of the parameter $d$ (2 *μ*m -16 *μ*m). To calculate the mode volume, we use a PML-based normalization approach, which is efficient and accurate for arbitrary geometries and dispersive materials^9,10^.

$$\begin{aligned} \left\langle{\tilde{\boldsymbol{E}}}_{m}\left( \boldsymbol{r} \right) \right|\left. {\tilde{\boldsymbol{E}}}_{m}\left( \boldsymbol{r} \right) \right\rangle=\iiint_{\Omega\cup\Omega_{PML}} \left[ {\tilde{\boldsymbol{E}}}_{m}\cdot\frac{\partial\omega\varepsilon}{\partial\omega}{\tilde{\boldsymbol{E}}}_{m}-{\tilde{\boldsymbol{H}}}_{\boldsymbol{m}}\cdot\frac{\partial\omega\varepsilon}{\partial\omega}{\tilde{\boldsymbol{E}}}_{m} \right]d^{3}\boldsymbol{r}\#\left( S9 \right) \end{aligned}$$

The integration domain of the inner product includes both general regions and the PML region. In order to describe the spatial extension and isotropic orientation distribution of crystalline grains in spin-coated MAPbI₃ perovskite thin films, the effective mode volume is defined as

$$\begin{aligned} \frac{1}{\tilde{V}_{m}}=Max\left( \frac{2\varepsilon_{0}n^{2}\left| \tilde{E}\left( r \right) \right|^{2}}{\left\langle\tilde{E}\left( r \right) \right|\left. \tilde{E}\left( r \right) \right\rangle} \right)\#\left( S10 \right) \end{aligned}$$

The structure is placed in a medium with a refractive index of 1.5, employing periodic boundary conditions, and the metal is described using the Drude model. To achieve stable and accurate results, the mesh in the spacing region between the two C-shaped structures is additionally refined and fixed.

Figure S7a quantitatively establishes the inverse proportionality between mode volume $V_{\mathrm{eff}}$​ and interlayer spacing *d*, showing a 62% reduction in $V_{\mathrm{eff}}$​ as *d* decreases from 16 μm to 2 μm. This trend aligns with the *d*-dependent variation in Rabi splitting intensity shown in Figure S7b.

Naturally, when the spatial distribution of modal fields remains stable, a denser structure enhances energy localization, leading to a reduced mode volume (V). The coordinated $V_{\mathrm{eff}}$​-${\hbarΩ}_{R}$​ modulation confirms mode-volume engineering as the dominant Rabi splitting enhancement mechanism in our BIC-phonon platform.


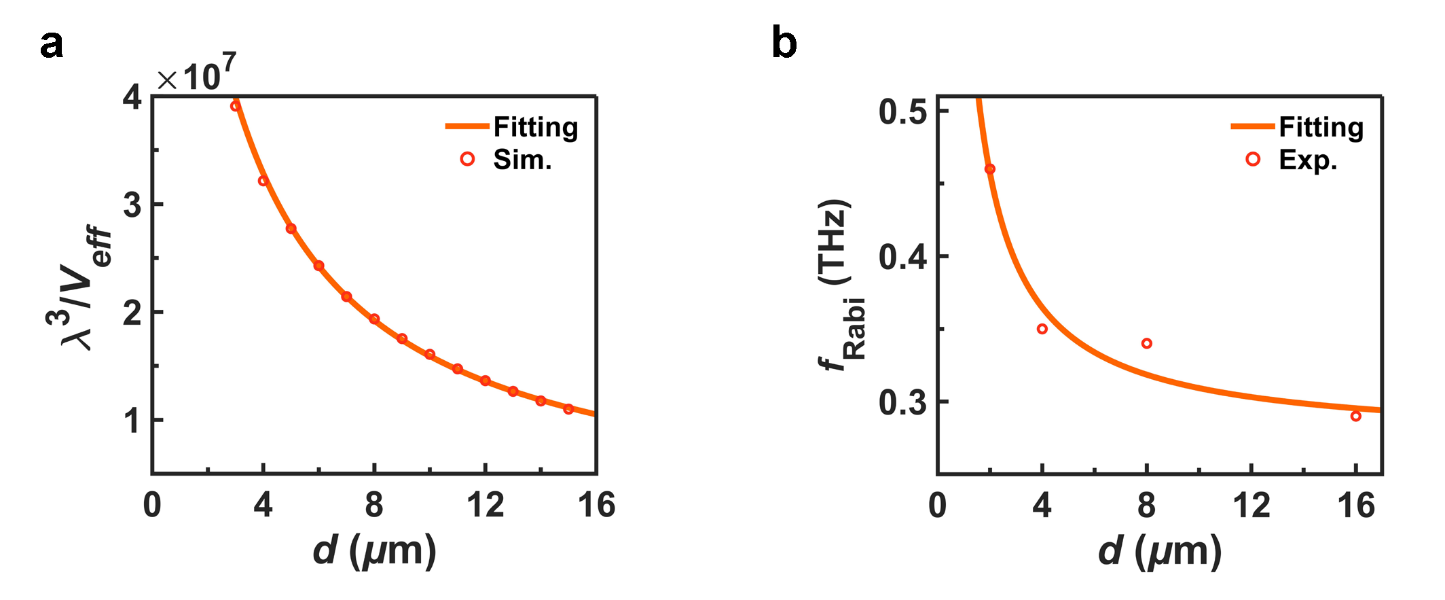


**Figure S7 | a,** Numerically calculated relationship between effective mode volume ($V_{\mathrm{eff}}$) and parameter *d*, demonstrating inverse proportionality. $\lambda$ is the resonance wavelength of eigenmode. **b,** Experimentally measured correlation between Rabi splitting and parameter *d*, showing monotonic enhancement of splitting magnitude with decreasing *d*.

**Supplementary Note 5: Rabi Splitting at Different BIC Asymmetry**

To investigate the influence of BIC asymmetry on Rabi splitting, a series of samples with different degrees of structural asymmetry $\Delta L$ are simulated and fabricated. The resonance of the BICs is aligned with that of the phonon by adjusting the scaling factor *S.* From the results in Figure S8b, it is evident that at low asymmetry levels, the coupling is significantly affected by the presence of another electric dipole mode in the structure, making it difficult to discern whether the BICs and phonons are coupled. However, when the asymmetry reaches $\Delta L$ = 9 *μ*m, the Rabi splitting of the phonon-polariton becomes clear and is unchanged as the degree of asymmetry increases. This behavior can be attributed to the fact that the intrinsic losses of the metal are substantial at low asymmetry, which means that reducing the degree of asymmetry does not significantly enhance the localized electric field strength in the metallic metasurfaces. Compared to PEC, the enhancement of the electric field in the Au metasurfaces is strongly limited by intrinsic losses. Therefore, due to the intrinsic losses associated with the metal, reducing the asymmetry of the BIC structures does not lead to a significant enhancement in Rabi splitting, but the criteria of strong coupling^11^ $c=2g/\sqrt{(\gamma_{\mathrm{BIC}}^{2} + \gamma_{\mathrm{Ph}}^{2})/2}$ is turned from 1.40 to 2.24.


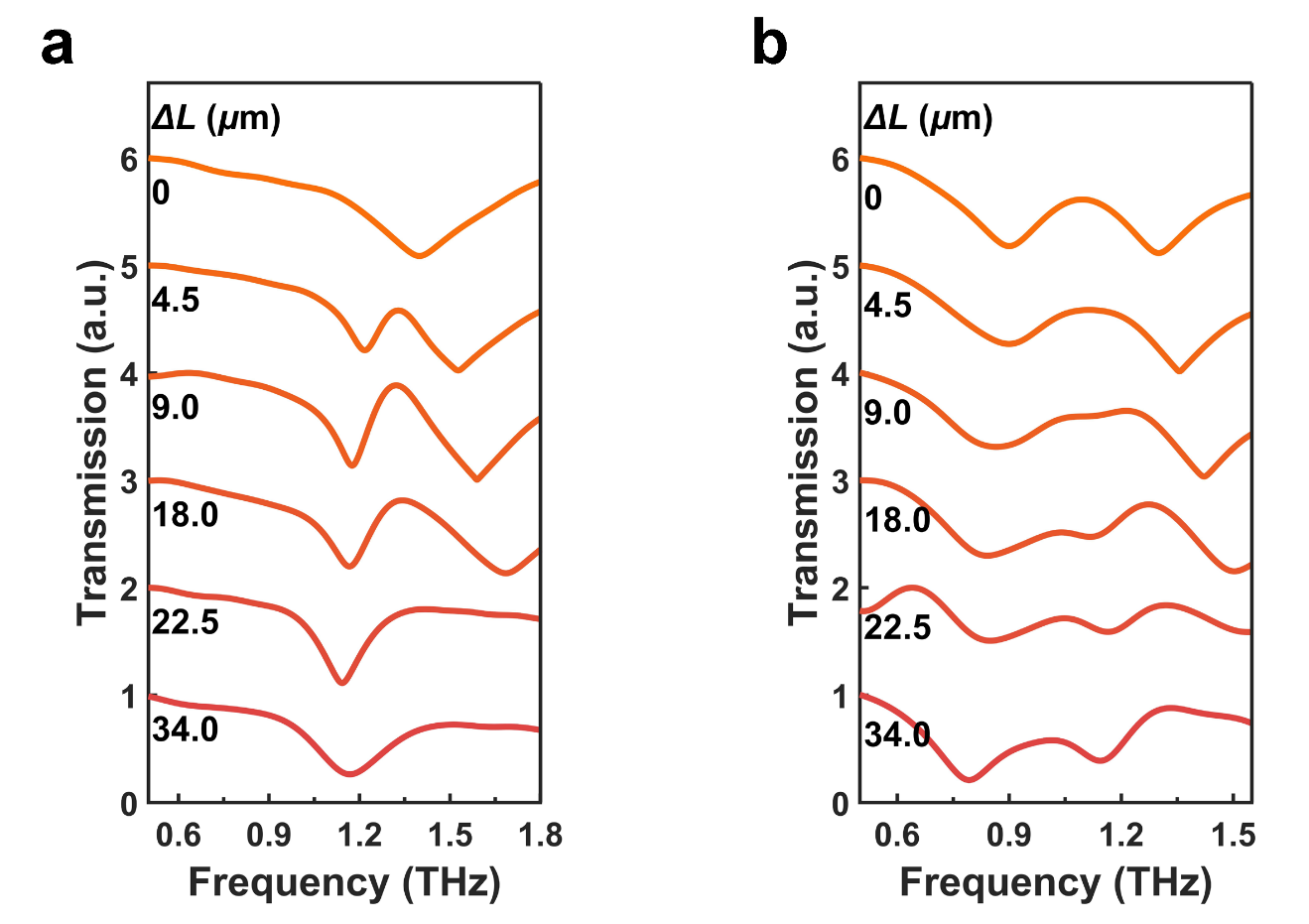


**Figure S8 | a-b,** Transmission amplitude of the metasurfaces under different symmetry-breaking conditions in the experiment. **(a)** without MAPbI_3_, **(b)** with MAPbI_3_.

**Supplementary Note 6:** **Impact of 1.85 THz Phonon Mode of MAPbI_3_ and Parasitic BIC Cavity Modes**

**(1) 1.85 THz Phonon Mode**:

Notably, Figure S6b reveals a notable discrepancy between the theoretically predicted upper polariton branch frequency derived from the full Hamiltonian and experimental observations under strong coupling conditions ($d$=2 *μ*m, measured Rabi splitting $\Omega_{R}=$ 0.46 THz). This inconsistency suggests the presence of additional coupling mechanisms beyond our initial model. Detailed analysis indicates significant perturbation from the 1.85 THz phonon mode, which exhibits the spectral overlap with the coupled system.

To address the potential influence of the 1.85 THz phonon mode, we expanded our theoretical framework to include a three-mode coupling model. The Hamiltonian now accounts for the BIC mode (*a*), the 0.95 THz phonon (*b*), and the 1.85 THz phonon (*c*):

$$\begin{aligned} \hat{H}=\hat{H}_{\mathrm{BIC}}+\hat{H}_{Ph1}+\hat{H}_{Ph2}+\hat{H}_{int1}+\hat{H}_{int2}+\hat{H}_{dia1}+\hat{H}_{dia2}\#\left（ S11 \right） \end{aligned}$$

where $g_{1}$​ and $g_{2}$​ are coupling strengths between the BIC mode and the two phonons. Diagonalizing this Hamiltonian via a 6×6 matrix $\mathbf{M}$, where$\mathbf{M}\vec{\mathbf{V}}=\omega\vec{\mathbf{V}}$ and $\det\left| \mathbf{M-}\omega\mathbf{I} \right|=0$, yields the eigenvalues $\omega_{1,2,3}$, reveals three polariton branches (upper, middle, lower).

As shown in Figures S9a, b, there is negligible differences between the uncoupled and coupled cases for the experimental data at $d$ =16 *μ*m ($\Omega_{R}=$0.28 THz) with the three-mode coupling. This confirms that the 1.85 THz phonon has a minimal impact on the coupling between the 0.95 THz phonon and BIC mode when the Rabi splitting $\Omega_{R}=$0.28 THz. In contrast, for  $d$ =2 *μ*m ($\Omega_{R}=$0.46 THz), Figures S9c, d demonstrate a significant compression of the experimentally observed Rabi splitting between the middle (MP) and lower polariton (LP) branches due to hybridization with the 1.85 THz phonon. The revised three-mode Hamiltonian, incorporating both phonons and the BIC mode, aligns quantitatively with the measured spectral features, highlighting the necessity of accounting for multimode effects in the ultrastrong coupling regime. However, this case still does not match the actual situation. Therefore, a more in-depth discussion is presented in the next section.


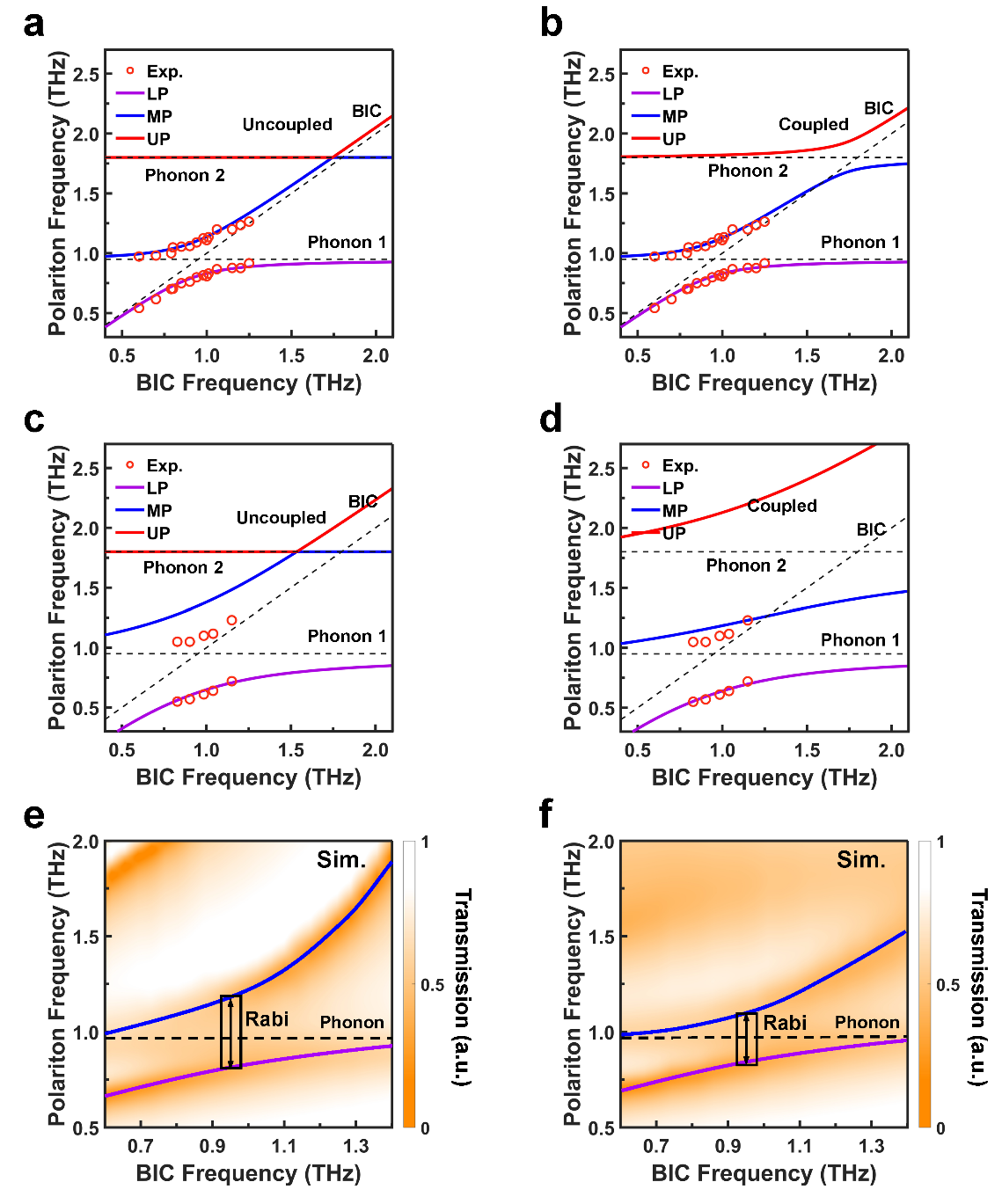


**Figure S9 |** **a-b,** Three-mode coupling model under *d* = 16 *μ*m ($\Omega_{R}=$0.28 THz): **(a)** Polariton frequency with phonon 2 uncoupled ($g$ = 0), showing three branches: upper (red, $\omega_{\mathrm{UP}}$ ), middle (blue, $\omega_{\mathrm{MP}}$ ), and lower (purple, $\omega_{\mathrm{LP}}$ ). **(b)** Inclusion of phonon 2 coupling ($g$ = 0.15$\omega_{ph2}$), splitting the upper and middle branch into hybridized states, while preserving the anti-crossing curves at $\omega_{ph2}$= 1.85 THz. **c-d,** Three-mode coupling model under *d* = 2 *μ*m ($\Omega_{R}=$ 0.46 THz): **(c)** Polariton frequency with phonon 2 uncoupled ($g$ = 0), yields Rabi splitting = 0 between upper (red) and middle (blue) bunches. **(d)** Stronger phonon 2 interaction ($g$ = 0.3$\omega_{ph2}$) induces spectral repulsion between middle and upper branches, with Rabi splitting 0.46 THz at $\omega_{ph1}$= 0.95 THz in experiment (red dots). **e-f**, Simulated transmission amplitude of BIC-MAPbI_3_ metasurfaces for different set of Lorentz oscillators. **e,** single (0.95 THz) Lorentz oscillators. **f,** dual (0.95 + 1.85 THz) Lorentz oscillators.

At zero detuning (BIC resonance aligned with the 0.95 THz phonon), the 1.85 THz phonon exhibits negligible coupling to the BIC mode (Figures S9e, f). However, when the BIC resonance approaches the 1.85 THz phonon frequency, the upper polariton branch shifts downward due to hybridization. Notably, theory and simulations incorporating both phonons yield Rabi splitting values closer to experimental observations (Figures S9b, d), confirming the necessity of this refinement.

The simulations presented in this paper employ a frequency domain solver, primarily based on the wave optics module of COMSOL Multiphysics. The structural parameters used in the simulations, as illustrated in Figures S9e, f, are identical to those utilized in the experimental configurations. The geometrical unit cell parameters are: $P_{x}$ = 66 *μ*m, $P_{y}$ = 32 *μ*m, $L_{x}$= 50 *µ*m, $L_{y}$= 25 *μ*m, $L_{1}$= 39.5 *μ*m, $m$ = 5 *μ*m, $w$ = 5 *μ*m, $h$ = 200 nm. The metal conductivity is set to 𝜎 = 6 $\times$ 10^7^ S m^-1^.

**(2)** **Higher-order BIC Cavity Mode**:

While the metasurface does supports a higher-order mode near 1.85 THz when the primary BIC model near 0.60 THz (Figure 2a), we had optimized structural parameters (the length $L_{y}$) to shift this mode beyond our experimental frequency range (0.6–1.3 THz) before the experiment as shown in Figure S10a. This ensures that the observed Rabi splitting (Figure 3a) arises solely from our BIC-phonon coupling system at zero detuning. As shown in Figure S8, when the higher-order cavity mode approaches the BIC resonance frequency, it induces a measurable reduction in Rabi splitting due to hybridization. To mitigate this, we systematically optimized the metasurface geometry during the design phase and set $L_{y}$= 32 *μ*m, ensuring minimal spectral overlap between the BIC mode and parasitic resonances. To further validate the negligible influence of higher-order BIC modes on the coupling system, we replaced the 1.85 THz phonon with a distant higher-order BIC mode ("Model 2") in the analysis for the $d$= 2 *μ*m case ($\Omega_{R}=$0.46 THz). Model 2, with a frequency detuned by ∼1.5 THz from the primary BIC resonance, was incorporated into the three-mode Hamiltonian. As shown in Figure S10b, the eigenfrequency of Model 2 exhibits no measurable shift, confirming that its coupling to the 0.95 THz phonon-BIC system is negligible due to the large spectral separation. This result demonstrates that the higher-order BIC modes do not perturb the primary polariton dynamics in this case. These findings are consistent with the experimental data and highlight the robustness of our mode-selective coupling design.


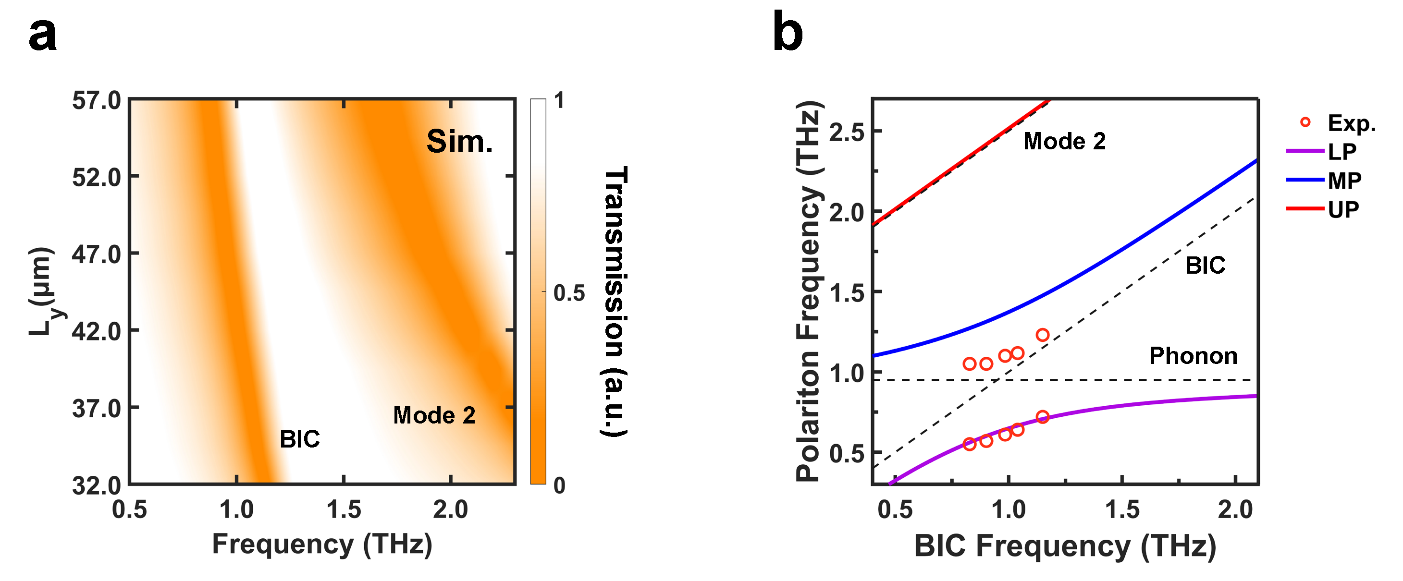


**Figure S10 |** **a,** Simulated transmission amplitude of the BIC resonances as a function of the parameter $L_{y}$, with other structural parameters fixed. When $L_{y}$= 32 *μ*m, the higher-order mode of BIC resonances was shifted beyond our experimental frequency range. **b,** Three-mode coupling framework incorporating a higher-order BIC mode (Model 2), base BIC mode, and primary phonon mode ($\omega_{ph1}$= 0.95 THz): With a designated phonon-Model 2 coupling strength of $g$ = 0.3$\omega_{ph1}$, spectral analysis reveals complete overlap between the upper polariton branch (red curve,) and Model 2 resonance (top dashed line). Consequently, Model 2 exhibits negligible participation in the target coupling dynamics and is excluded from further analysis.

**(3) 1.85 THz Phonon Mode and Higher-order BIC Cavity Mode**:


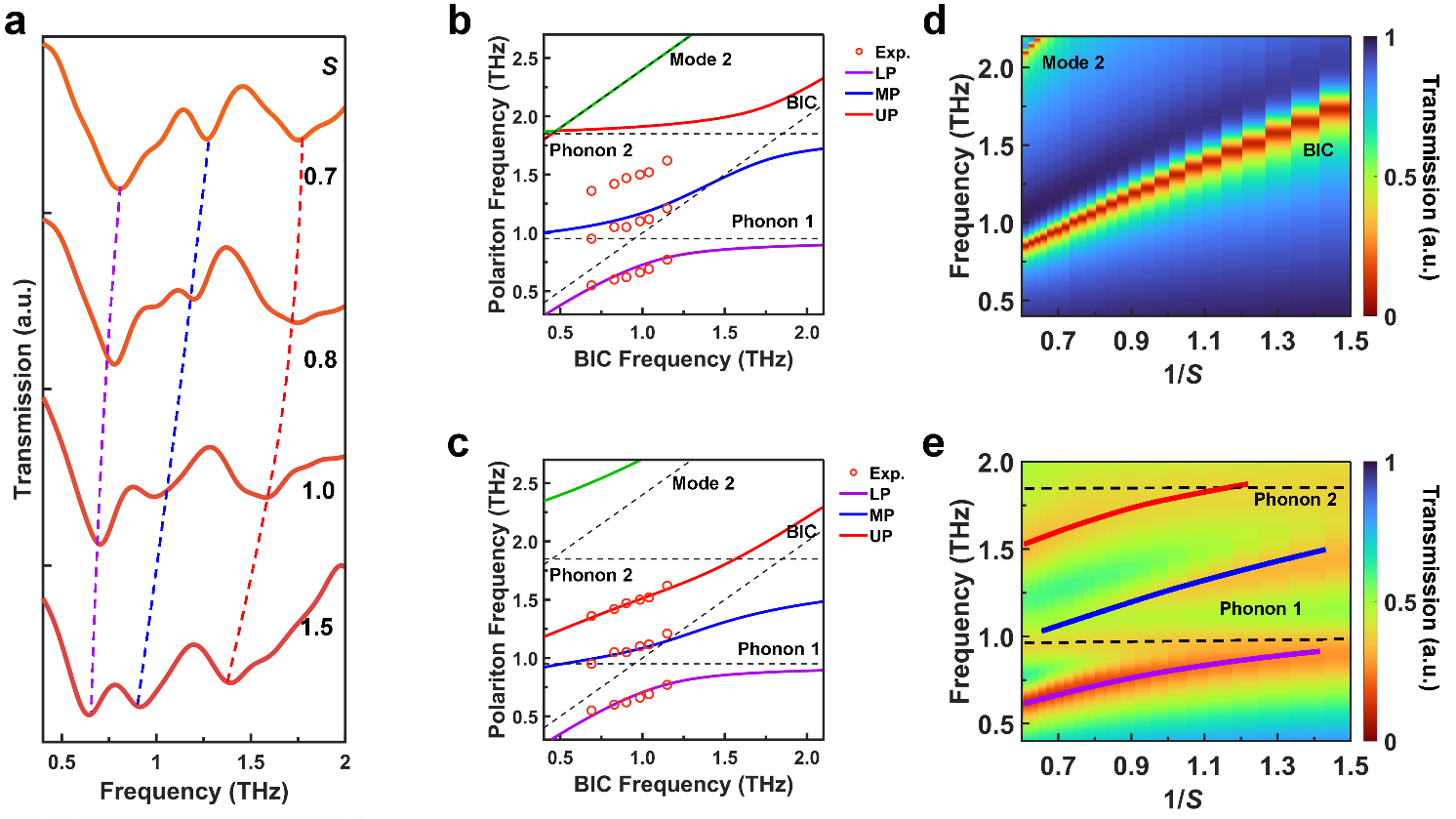


**Figure S11 | a,** Measured transmission amplitudes of BIC resonances across the frequency of perovskite phonon tuned via scaling the in-plane geometric parameters. The new parameters were derived by multiplying *S* with the in-plane geometric parameters. **b-c,** Four-mode coupling model under *d* = 2 *μ*m ($\Omega_{R}=$ 0.46 THz): **(b)** Polariton frequency with high order modes uncoupled ($g$ = 0), yields Rabi splitting = 0 between mode 2 and phonon 2. **(c)** Stronger high order modes interaction ($g$ = 0.30$\omega_{ph2}$) induces spectral repulsion between mode 2 and phonon 2, with Rabi splitting 0.46 THz at $\omega_{ph1}$= 0.95 THz in experiment (red dots). **d,** Simulated transmission amplitude of the BIC resonances as a function of the parameter $S$, with other structural parameters fixed. **e**, Simulated transmission amplitude of BIC-MAPbI_3_ metasurfaces for dual (0.95 + 1.85 THz) Lorentz oscillators.

To ensure rigorous analysis, we fabricated and characterized several samples, with particular attention to the spectral region around 1.85 THz. Figure S11a presents four representative transmission spectra, where we focused on the reliable sub-2 THz spectral range due to experimental constraints. We developed a comprehensive four-mode coupling model that explicitly accounts for the interaction between the metasurface's higher-order mode and the high-frequency phonon mode. As shown in Figure S11b-c, our model successfully reproduces the experimental observations, demonstrating that this higher-order coupling significantly influences the system's optical response. Furthermore, Figures S11d-e present numerical simulations comparing systems with and without the phonon modes. The simulations clearly reveal three distinct polariton branches within our experimental spectral window, corresponding to the multi-mode coupling features observed in our measurements (Figure S11a). These results provide compelling evidence for the importance of including all relevant coupling channels in our theoretical framework.

**Supplementary Note 7: Wavelet Transform**

The wavelet transform is a powerful time-frequency analysis tool that overcomes the inherent trade-off between temporal and spectral resolution in conventional Fourier transforms. Unlike Fourier analysis, which decomposes a signal into infinite sinusoidal waves, wavelet transform uses localized basis functions ("wavelets") scaled and shifted across the time domain. This enables precise resolution of transient features in non-stationary signals like THz pulses. We employed the generalized Morse wavelet^11^. The generalized Morse wavelet in the Fourier domain is^12^

$$\begin{aligned} \psi_{\beta,\gamma}\left( \omega\right)=U\left( \omega\right)a_{\beta,\gamma}\omega^{\beta}e^{-\omega^{\gamma}}\#\left( S12 \right) \end{aligned}$$

where $U(\omega)$ is the unit step function, $a_{\beta,\gamma}$ is a normalizing constant, $\beta$ controls the time-domain decay rate (related to compactness), and $\gamma$ characterizes the symmetry of the Morse wavelet. The time-bandwidth product $P^{2}=\beta\gamma$ determines the wavelet duration in time.

At $\gamma=3$, Morse wavelets achieve minimum Heisenberg area (optimal joint time-frequency resolution) and zero demodulate skewness (symmetric envelope)^13^. Reducing *β* increases temporal resolution but sacrifices spectral precision (Figure S12). Therefore, we selected $\gamma=3, \beta=60$ to balance the detection of rapid phonon dynamics (~ 1 ps) and spectral resolution (~ 0.1 THz) for the 0.95 THz phonon mode. The Morse wavelet transform with *β* = 60 was selected for analyzing terahertz time-domain data (Figures S12c, d). Compared with short-time Fourier transform results, this wavelet decomposition demonstrates reduced spectral artifacts, enhanced precision in mode resolution, and superior suitability for investigating temporal evolution of coupled modes.


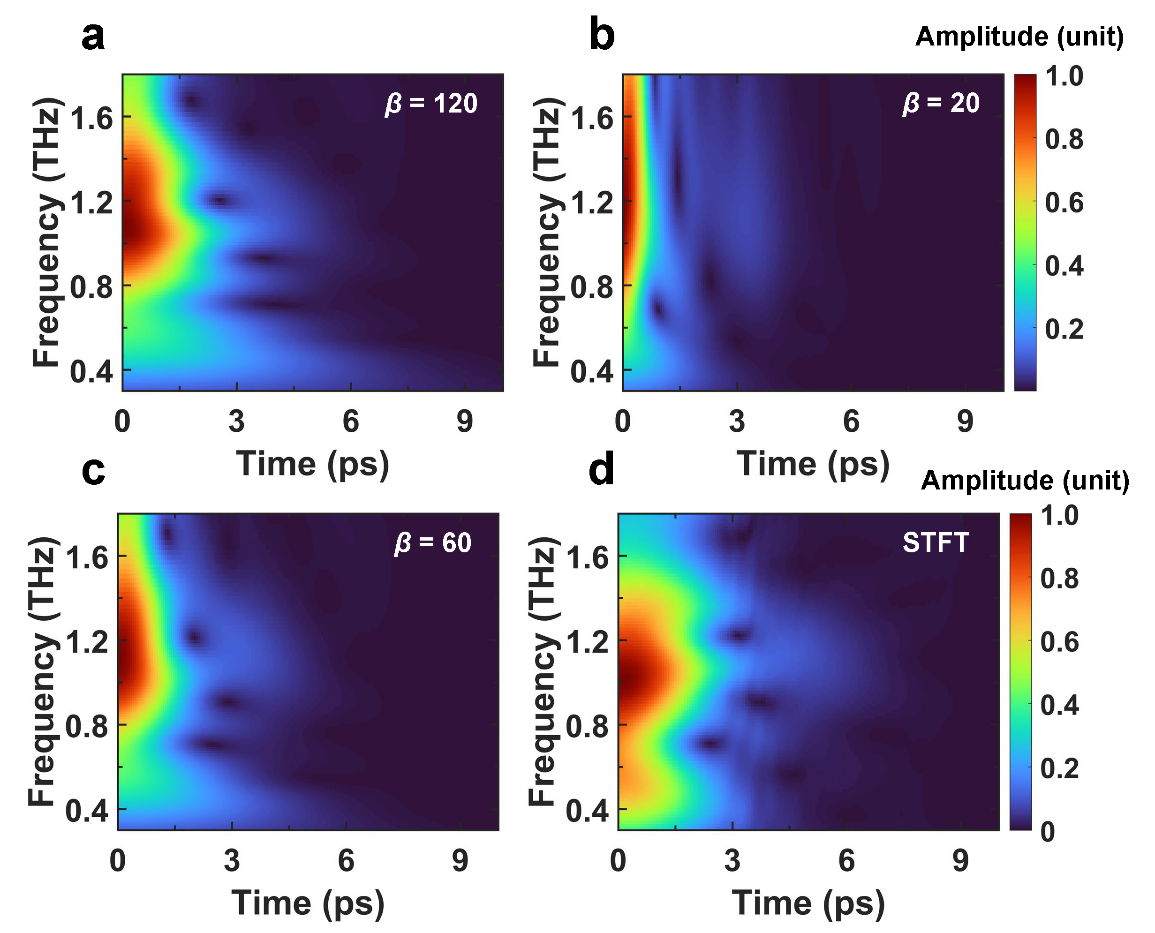


**Figure S12 |** Wavelet analysis of terahertz time-domain waveforms transmitted through the $d$ =2 *μ*m sample using varying *β* parameters and the short-time Fourier transform result. The analysis reveals a fundamental trade-off in time-frequency resolution: increasing *β* values enhance spectral resolution ($\Delta f \propto1/\beta$) while concurrently degrading temporal resolution ($\Delta t \approx1/(2\pi\Delta f)$), consistent with the Heisenberg-Gabor uncertainty principle governing wavelet transforms. **a,**$\beta=120$, **b,**$\beta=20$, **c,**$\beta=60$ **d,** Control result based on the short-time Fourier transform (STFT).

Figures S13a and d illustrate the time-domain waveforms of the incident and transmitted THz waves. The frequency domain spectra in Figures S13b and e are obtained through a fast Fourier transform (FFT), clearly revealing the frequencies and absorption characteristics of the two modes of the phonon-polaritons. However, the Fourier transform does not reflect the frequency distribution of the THz waves at different times. In contrast, the time-frequency distribution obtained through wavelet transform, shown in Figures S13c and f, provides a clear representation of the frequency distribution at different times. Especially, the time-frequency distribution in Figure S13f allows for the observation of the phonon mode at 0.95 THz, which is difficult to discern in the frequency spectrum from the Fourier transform in Figure S13e.

Raw THz signals in Figures S13a, d were normalized to a reference pulse (vacuum) to remove system artifacts. The preprocessed signal $E(t)$ was convolved with Morse wavelets scaled across 0.2–2.0 THz^11^:

$\begin{aligned} W\left( t,\omega\right)=\int_{-\infty}^{\infty} E\left( \tau\right)\cdot\frac{1}{\sqrt{s}}\Psi*\left( \frac{\tau-t}{s} \right)d\tau\#\left( S13 \right) \end{aligned}$

where $s=C_{1}^{\gamma}\left| 2\pi\left| f \right| \right|^{-\gamma}=\Delta\omega/\omega_{0}$ is the scale parameter. The acquired time-frequency spectrum shows the dynamic processes of both the upper/lower energy branches in the coupled system and their associated phonon modes (Figures S13c, f). While FFT provides high spectral resolution (Figures S13b, e), it obscures transient dynamics. As shown in Figure S13f, wavelet analysis reveals the upper/lower energy branches emerging at *t* ≈1.5 ps, aligning with BIC-phonon energy exchange. This transient feature is inseparable in FFT spectra due to limited time localization.


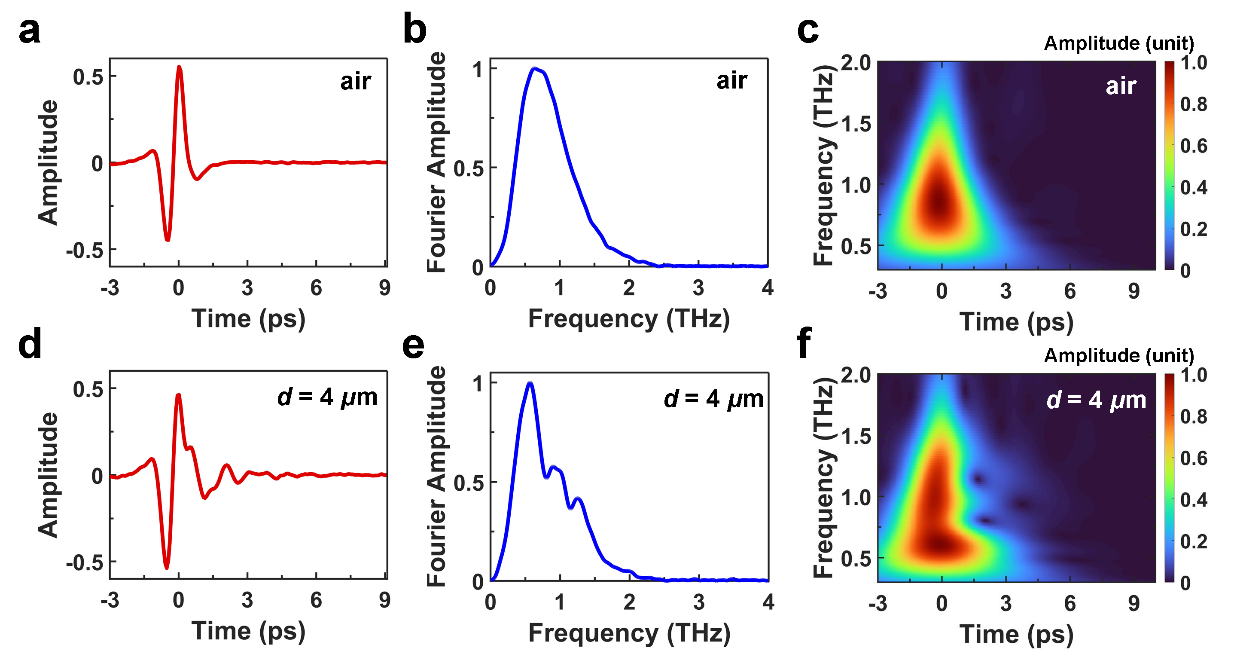


**Figure S13 | a,** The profile of the terahertz field without a sample. **b,** The spectrum of the terahertz through direct Fourier transform from the terahertz field in **(a). c,** The time-frequency spectrum obtained through wavelet transform from the terahertz field in **(a)**. **d,** The terahertz field transmitted through a sample with a parameter of $d$ = 4 *μ*m. **e,** The spectrum of the terahertz through direct Fourier transform from the terahertz field in **(d)**. **f,** The time-frequency spectrum obtained through wavelet transform from the terahertz field in **(d)**. (All data have been normalized)


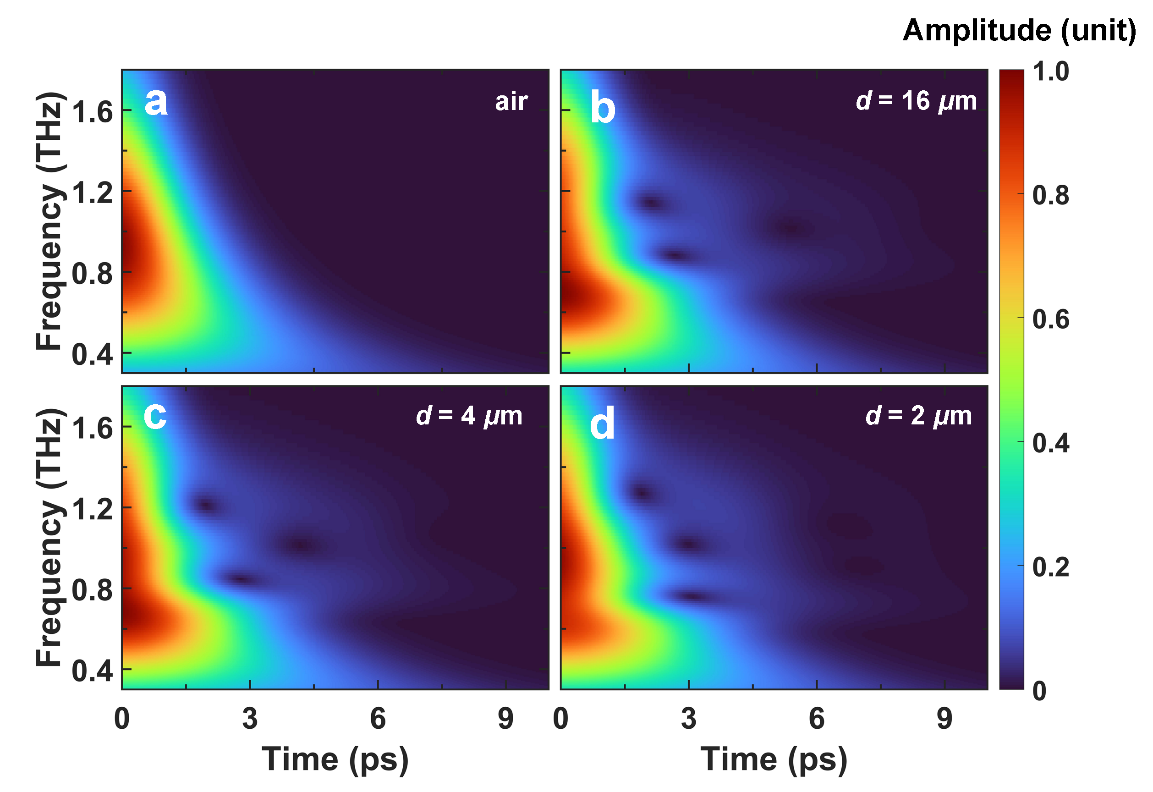


**Figure S14 | a-d,** Time-evolved spectrum was obtained through the wavelet transform method. **(a)** without samples, **(b)** $d$ = 16 *μ*m, **(c)** $d$ = 4 *μ*m, **(d)** $d$ = 2 *μ*m.

**Supplementary Note 8: Evolution of Phonon-polariton**

Figure S15a displays the time-frequency domain representation for $d$ = 4 *μ*m. By extracting the time evolution of the amplitude information at 𝜔 = 0.95 THz, we obtain the wavepackets shown in Figure S15b. In wavepacket 1, the terahertz field does not interact with the material, while the wavepacket 2, the terahertz field is associated with phonon re-emission predominates. The amplitude of the wavepacket 2 characterizes the intensity of phonon radiation. By extracting the amplitude of the electric field, we can obtain the intensity and phase variations of the terahertz field at 𝜔 = 0.95 THz shown in Figure 4 in the main text. The timing of the intensity and phase discontinuities coincide with the timing of the phonon wavepacket alternation, illustrating the process of phonon absorption and re-emission of terahertz radiation. Using the same method, the electric field evolution at the upper and lower branches of the phonon-polaritons can also be extracted in Figure S16.


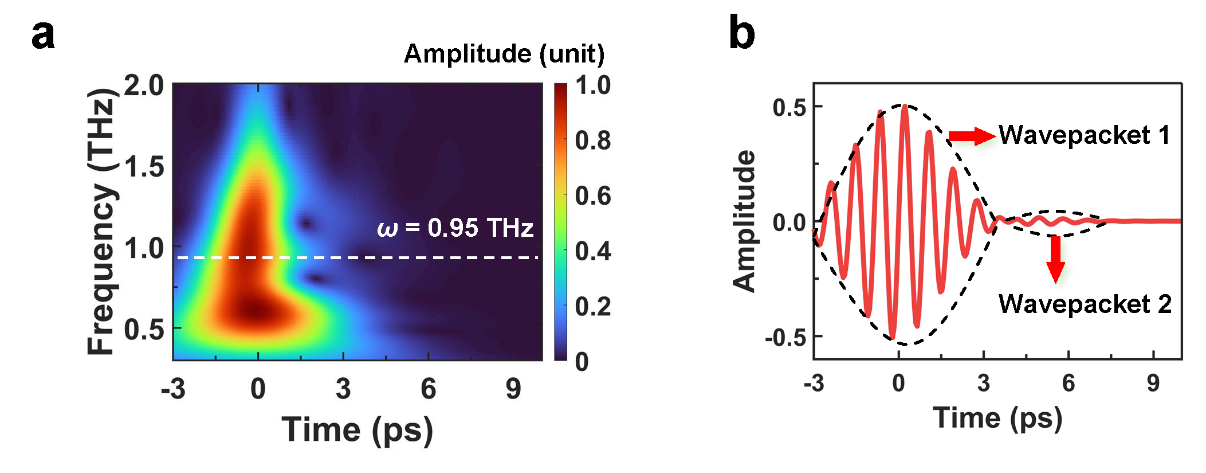


**Figure S15 | a,** The time-frequency domain representation of the terahertz field transmitted through the sample with $d$ = 4 *μ*m. The white dashed line indicates the position of the phonon at 𝜔 = 0.95 THz. By extracting the terahertz field evolution information along the white dashed line, the mode evolution is presented in **(b)**. **b,** The red curve represents the evolution of the terahertz field at 𝜔 = 0.95 THz, while the black dashed lines outline two wavepackets.


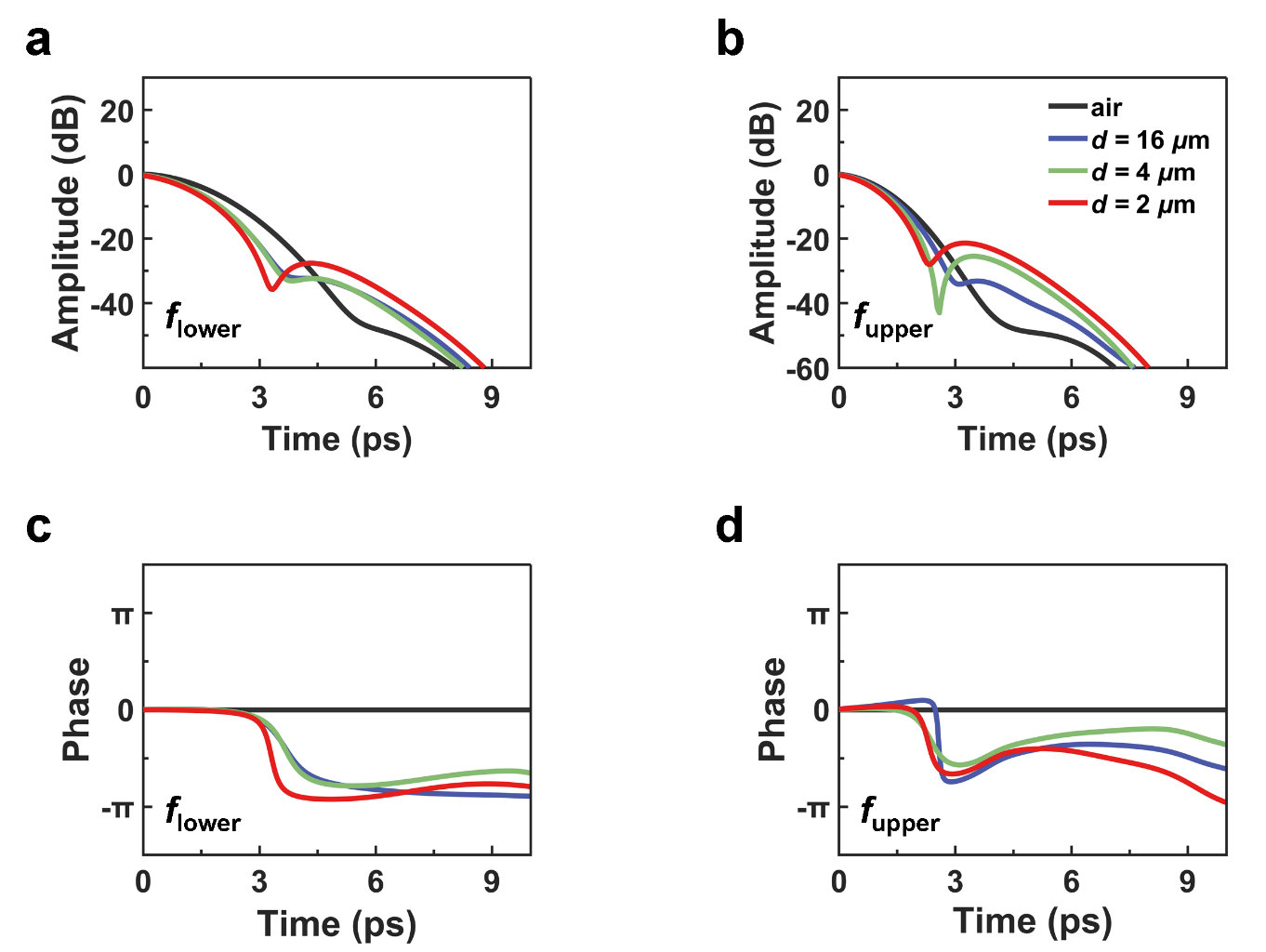


**Figure S16 |** Time evolution of the intensity and phase of the terahertz field for different parameters$d$. **a,** The intensity evolution of the lower polariton branch. **b,** The intensity of the upper polariton branch. **c,** The phase evolution of the lower polariton branch. **d,** The phase evolution of the upper polariton branch.

We extracted the upper and lower polariton branches from Figures S14 for further analysis of intensity and phase. As shown in Figures S16c-d, a clear phase shift of 𝜋 is observed, leading to the destructive interference of the far-field radiation at the frequency of upper and lower polariton branches. The nearly simultaneous changes of the intensity and phase with respect to time indicate a strong correlation between the upper and lower polariton branches.

Furthermore, we have conducted a temperature-dependent experiment to systematically investigate the correlation between phonon re-emission intensity and the amplitude of wavepacket 2. Previous studies^14^ have demonstrated that reduced thermal phonon dissipation at lower temperatures enhances the oscillator strength in perovskite materials. Building on this foundation, we designed a cryogenic experiment to measure terahertz time-domain signals of pure perovskite thin films across a temperature range of 4 K–300 K. In Figure S17a, transmission spectroscopy analysis revealed a progressive enhancement in the 0.95 THz absorption peak intensity accompanied by spectral narrowing (reduced full-width-at-half-maximum) as temperature decreased. This behavior aligns with the Lorentz oscillator model, confirming the temperature-dependent enhancement of oscillator strength. The strengthened oscillator strength directly corresponds to amplified far-field phonon re-emission, as evidenced by our experimental observations.

Time-frequency analysis through continuous wavelet transform further resolved two distinct wavepackets in the time-domain signals. As illustrated in Figure S17e, the intensity of wavepacket 2 exhibited a pronounced positive correlation with decreasing temperature, mirroring the temperature evolution of phonon re-emission intensity derived from transmission spectra. This consistent thermal response unambiguously establishes that the post-transition wavepacket originates predominantly from phonon re-emission processes. The combined evidence from spectroscopic characterization and time-frequency analysis provides conclusive support for the stated conclusion.


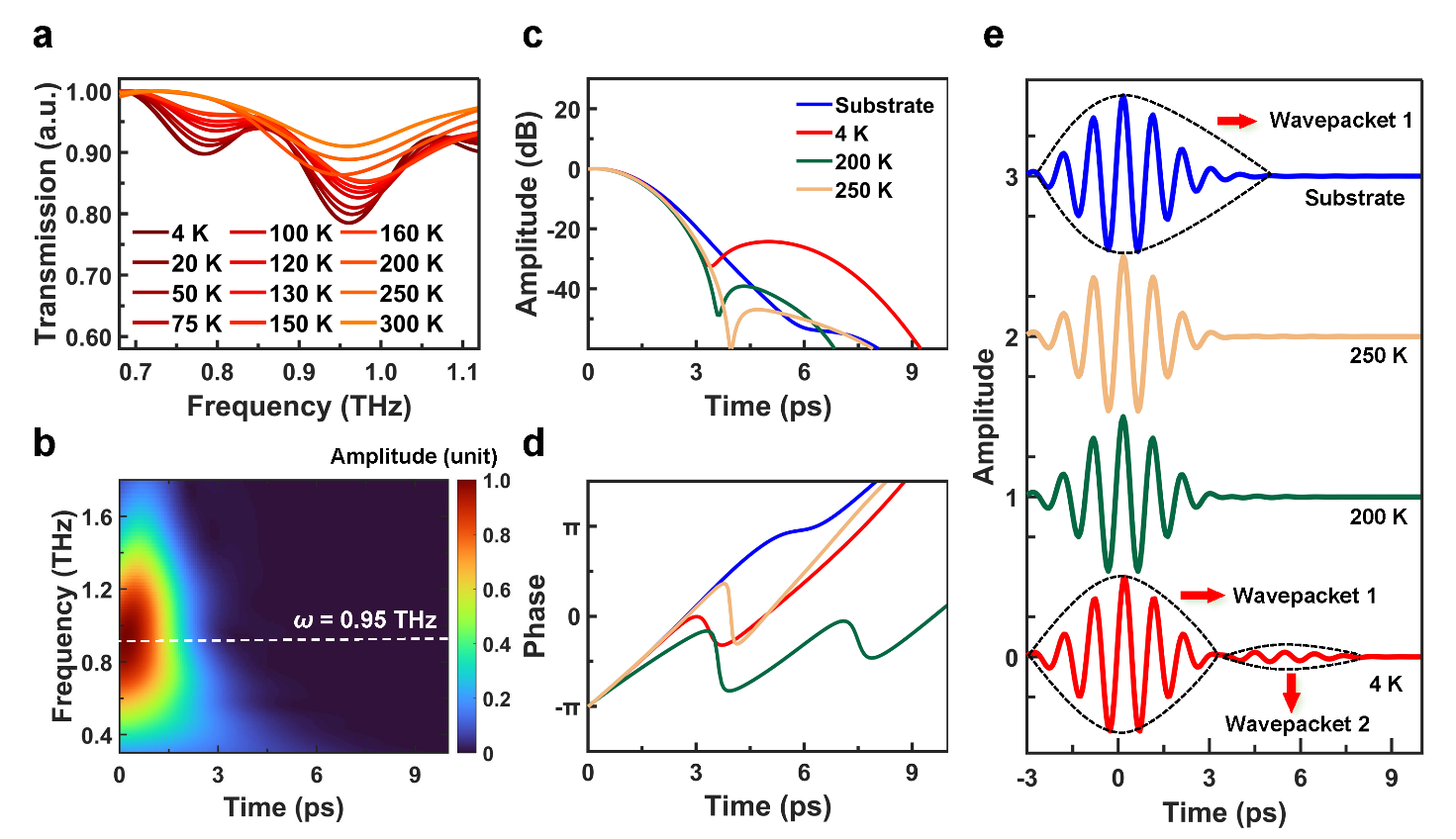


**Figure S17 |** **a,** Temperature-dependent phonon response of MAPbI₃ at 0.95 THz: Progressive cooling from 300 K to 4 K reduces the phonon linewidth (FWHM: 0.20 THz → 0.12 THz) while deepening absorption depth (transmittance: 0.92 → 0.78), consistent with enhanced oscillator strength predicted by Lorentzian modeling. Concurrent emergence of a 0.77 THz phonon mode indicates temperature-activated structural phase transitions of MAPbI₃.**b,** Time-frequency analysis of perovskite films via Morse wavelet transform: The pseudo-color map visualizes mode dynamics, where the horizontal axis represents time delay (0–10 ps) and the vertical axis denotes spectral components. **c-d,** Temperature-dependent mode evolution at $\omega$ = 0.95 THz: **(c)** Amplitude profiles exhibit dual-wavepacket structure, with Wavepacket 2 intensity increasing by 20 dB at 4 K compare with 300 K. **(d)** Phase discontinuities (π-jump) confirm phonon re-emission characteristics. (e) Reconstructed time-domain signals of $\omega$ = 0.95 THz at representative temperatures: Integrated amplitude-phase analysis reveals systematic enhancement of Wavepacket 2 (phonon re-emission component) with cooling.

**Supplementary Note 9:** **Sample Fabrication**


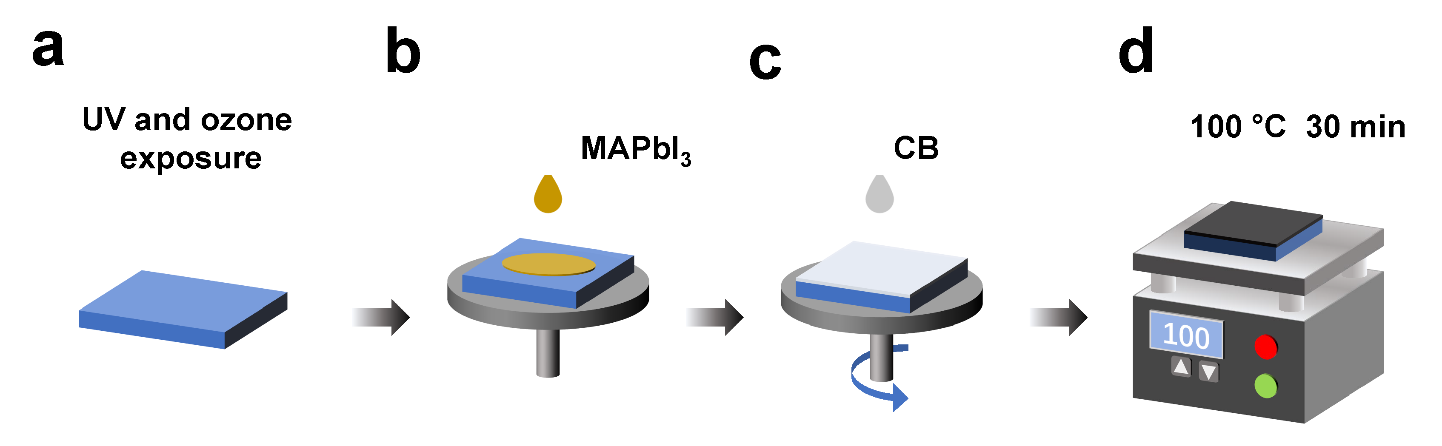


**Figure S18 |** Preparation steps for perovskite. **a,** The quartz substrate is initially cleaned using UV light and ozone exposure. **b,** After dropping the lead iodide perovskite precursor solution onto the substrate, it is spun at a speed of 6000 rpm to achieve a uniform film of lead iodide perovskite. **c,** During the spin-coating process, toluene solution is added to improve the crystallization of the perovskite film. **d,** After spin-coating, the samples undergo thermal annealing treatment.


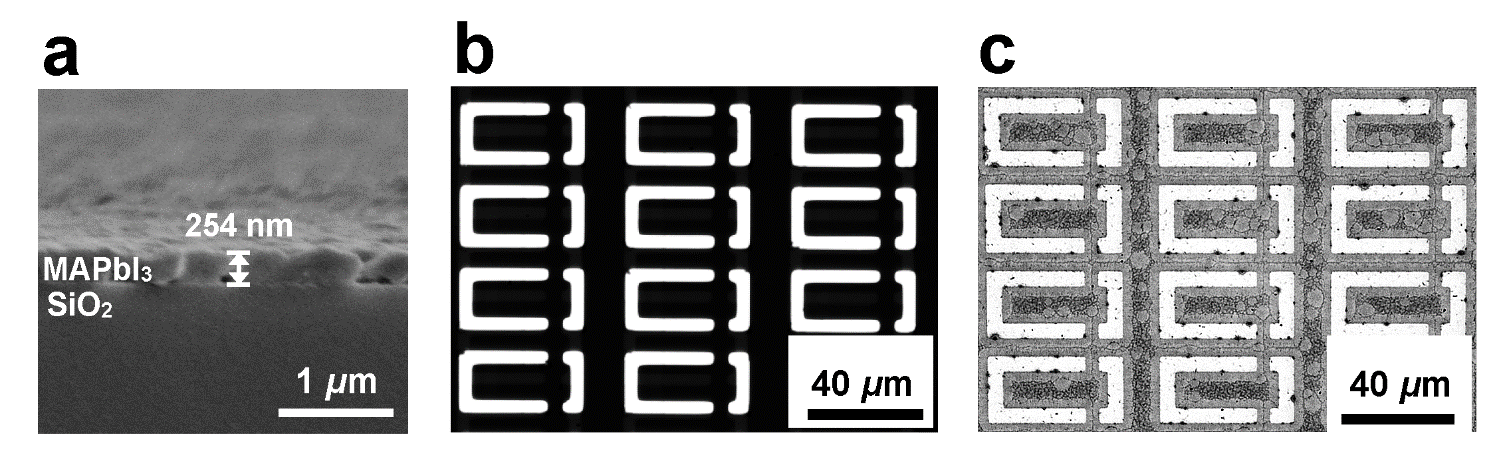


**Figure S19 | a,** MAPbI_3_ thin film was spin-coated onto a quartz substrate, and imaged using SEM (Scanning Electron Microscope). **b-c,** Optical images of the metasurfaces; **(b)** before and **(c)** after the spin-coating of MAPbI_3_.

**Supplementary Note 10: Experiment Setup**

The schematic of the experimental setup is shown in Figure S20. The THz-TDS system is driven by a Ti-sapphire femtosecond laser, which generates laser pulses with a duration of 35 fs, a repetition rate of 1 kHz, and a center wavelength of 800 nm. We utilize efficient metallic spintronic emitters of ultra-broadband terahertz radiation to generate terahertz pulses based on the principle of ultrafast photoinduced spin currents. The detection crystal used is ZnTe. To eliminate the influence of water molecules in the air on the experiment, the portion of terahertz propagation is filled with dry air.

**
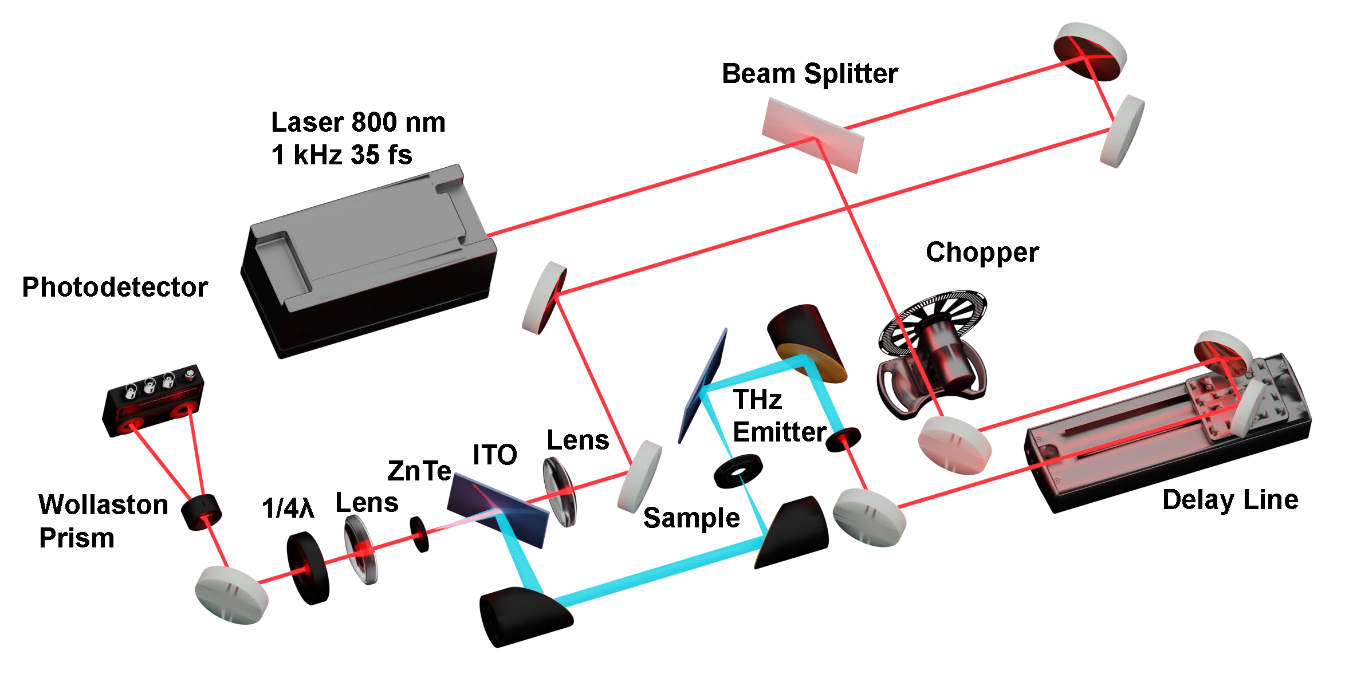
**

**Figure S20 |** Schematics of terahertz generation and detection.

**Reference**

1. Hsu, C. W., Zhen, B., Stone, A. D., Joannopoulos, J. D. & Soljačić, M. Bound states in the continuum. *Nat. Rev. Mater.* **1**, 16048 (2016).

2. Zhao, X. *et al.* Terahertz investigation of bound states in the continuum of metallic metasurfaces. *Optica* **7**, 1548 (2020).

3. Liang, Y., Tsai, D. P. & Kivshar, Y. From Local to Nonlocal High-Q Plasmonic Metasurfaces. *Phys. Rev. Lett.* **133**, 053801 (2024).

4. Koshelev, K., Lepeshov, S., Liu, M., Bogdanov, A. & Kivshar, Y. Asymmetric Metasurfaces with High-Q Resonances Governed by Bound States in the Continuum. *Phys. Rev. Lett.* **121**, 193903 (2018).

5. Sendner, M. *et al.* Optical phonons in methylammonium lead halide perovskites and implications for charge transport. *Mater. Horiz.* **3**, 613–620 (2016).

6. La-o-vorakiat, C. *et al.* Phonon Mode Transformation Across the Orthohombic–Tetragonal Phase Transition in a Lead Iodide Perovskite CH_3_NH_3_PbI_3_ : A Terahertz Time-Domain Spectroscopy Approach. *J. Phys. Chem. Lett.* **7**, 1–6 (2016).

7. Li, X. *et al.* Vacuum Bloch–Siegert shift in Landau polaritons with ultra-high cooperativity. *Nat. Photon.* **12**, 324–329 (2018).

8. Zhang, Q. *et al.* Collective non-perturbative coupling of 2D electrons with high-quality-factor terahertz cavity photons. *Nat. Phys.* **12**, 1005–1011 (2016).

9. Lalanne, P., Yan, W., Vynck, K., Sauvan, C. & Hugonin, J.-P. Light Interaction with Photonic and Plasmonic Resonances. *Laser Photon. Rev.* **12**, 1700113 (2018).

10. Wu, T., Arrivault, D., Yan, W. & Lalanne, P. Modal analysis of electromagnetic resonators: user guide for the MAN program. *Comput. Phys. Commun.* **284**, 108627 (2023).

11. Olhede, S. C. & Walden, A. T. Generalized Morse wavelets. *IEEE Trans. Signal Process.* **50**, 2661–2670 (2002).

12. Lilly, J. M. jLab: A data analysis package for Matlab. http://www.jmlilly.net/code (2024).

13. Lilly, J. M. & Olhede, S. C. Higher-Order Properties of Analytic Wavelets. *IEEE Trans. Signal Process.* **57**, 146–160 (2009).

14. Milot, R. L., Eperon, G. E., Snaith, H. J., Johnston, M. B. & Herz, L. M. Temperature-Dependent Charge-Carrier Dynamics in CH_3_NH_3_PbI_3_ Perovskite Thin Films. *Adv. Funct. Mater.* **25**, 6218–6227 (2015).
